# The mechanism underlying extrapulmonary complications of the coronavirus disease 2019 and its therapeutic implication

**DOI:** 10.1038/s41392-022-00907-1

**Published:** 2022-02-23

**Authors:** Qin Ning, Di Wu, Xiaojing Wang, Dong Xi, Tao Chen, Guang Chen, Hongwu Wang, Huiling Lu, Ming Wang, Lin Zhu, Junjian Hu, Tingting Liu, Ke Ma, Meifang Han, Xiaoping Luo

**Affiliations:** 1 National Medical Center for Major Public Health Events, Department and Institute of Infectious Disease, Tongji Hospital, Tongji Medical College, Huazhong University of Science and Technology, Wuhan, China; 2 National Medical Center for Major Public Health Events, Department of Pediatrics, Tongji Hospital, Tongji Medical College, Huazhong University of Science and Technology, Wuhan, China

**Keywords:** Infectious diseases, Respiratory tract diseases

## Abstract

The coronavirus disease 2019 (COVID-19) is a highly transmissible disease caused by the severe acute respiratory syndrome coronavirus 2 (SARS-CoV-2) that poses a major threat to global public health. Although COVID-19 primarily affects the respiratory system, causing severe pneumonia and acute respiratory distress syndrome in severe cases, it can also result in multiple extrapulmonary complications. The pathogenesis of extrapulmonary damage in patients with COVID-19 is probably multifactorial, involving both the direct effects of SARS-CoV-2 and the indirect mechanisms associated with the host inflammatory response. Recognition of features and pathogenesis of extrapulmonary complications has clinical implications for identifying disease progression and designing therapeutic strategies. This review provides an overview of the extrapulmonary complications of COVID-19 from immunological and pathophysiologic perspectives and focuses on the pathogenesis and potential therapeutic targets for the management of COVID-19.

## Introduction

The severe acute respiratory syndrome coronavirus 2 (SARS-CoV-2) is a highly contagious and pathogenic virus that was identified as a causative agent of the coronavirus disease 2019 (COVID-19).^1^ As of January 6, 2022, SARS-CoV-2 has infected nearly 289 million people and caused over 5.4 million deaths globally.^2^ Accumulating evidence suggests that SARS-CoV-2 infection primarily attacks the lung and causes respiratory diseases ranging from mild cold to more severe illness such as severe acute respiratory syndrome (ARDS), but it can also affect other organs and have systemic consequences with multiple organ injury.^3^ The extrapulmonary complications^4^ include a wide spectrum of disorders with cardiovascular,^5^ endothelial,^6^ coagulation,^7^ renal,^8^ hepatobiliary,^9^ gastrointestinal,^10^ endocrinological,^11^ neurological^12^ involvement, which may occur in severe and critically ill patients and are associated with prolonged hospitalization and increasing mortality risk. The extrapulmonary organ injury of COVID-19 may result from direct injury mediated by SARS-CoV-2 invasion, endothelial cell damage, or possible indirect mechanisms secondary to excessive local and systemic inflammatory responses. Angiotensin-converting enzyme 2 (ACE2)^13^ has been identified as the entry receptor for SARS-CoV-2. The widespread distribution of ACE2 across multiple organs and tissues makes the virus-mediated direct tissue damage a plausible mechanism of systematic injury.^14^ Moreover, dysregulated immune response, endothelial damage as well as thromboinflammation may also account for the extrapulmonary complications of COVID-19.^6,15^ In this review, we narratively summarized the published literature on extrapulmonary consequences of COVID-19, and provided a comprehensive perspective on the extrapulmonary organ-specific pathophysiology and potential therapeutic strategies for COVID-19, in order to help scientists and clinicians to identify and monitor the spectrum of disease, and to establish research priorities within this field.

## Pathogenesis of SARS-CoV-2 infection

Key mechanisms underlying pathophysiology of extrapulmonary organ injury secondary to SARS-CoV-2 infection include direct viral invasion, imbalance of renin–angiotensin-aldosterone system (RAS), dysregulation of the immune response, endothelial cell damage, and thromboinflammation. These mechanisms responsible for multiple organ involvement of COVID-19 has not yet been fully understood. ACE2-mediated virus entry and dysregulated RAS may be unique to SARS-CoV-2 infection, while immune dysregulation characterized by excessive release of proinflammatory cytokines and microcirculation disorder may occur in other critical conditions such as sepsis.

### Direct mechanism of SARS-CoV-2 infection

SARS-CoV-2 is an enveloped virus with a positive-sense single-stranded RNA (+ssRNA) genome of around 30-kb. A mature SARS-CoV-2 particle contains four main structural components, including spike (S), envelope (E), membrane (M) glycoproteins, and nucleocapsid phosphoprotein (N). The S glycoprotein mediates virus entry into target cells. E protein is a small integral membrane protein acting on viral assembly, budding, envelope formation, and pathogenesis.^16^ N protein is an abundantly expressed RNA-binding protein that plays a critical role in the replication, transcription, and genome packaging of SARS-CoV-2. M protein is key for the assembly of viral particles through interacting with all other structural proteins. These interactions between structural proteins help form replication-incompetent virus-like particles (VLPs), which resemble the morphological structure of SARS-CoV-2^17^ and are efficient platform for vaccine development.

SARS-CoV-2 can enter the host cells either via endocytosis or via direct fusion with the plasma membrane. The S protein binding to ACE2 represents the initial step of SARS-CoV-2 infection, thus it is the main target for the design of vaccines and inhibitors of viral entry. S protein includes S1 and S2 subunits. The S1 subunit comprises an N-terminal domain (NTD) and the receptor-binding domain (RBD).^18^ The RBD contains a conserved core and receptor-binding motif (RBM), which is a variable region of S protein responsible for direct binding to ACE2 and the key target of neutralizing antibodies.^19,20^ The S2 subunit mediates fusion of the viral envelope with host cellular membrane. It consists of a highly conserved fusion peptide (FP) domain, two heptad-repeat domains (HR1 and HR2), a central helix (CH), a connector domain (CD), transmembrane domain (TM), and cytoplasmic tail (CT).^21^

ACE2 was identified as the binding receptor of both SARS-CoV and SARS-CoV-2. The RBD of SARS-CoV-2 has a higher ACE2-binding affinity compared to that of SARS-CoV, supporting efficient cell entry.^22^ The enhanced affinity may increase the infectivity of SARS-CoV-2. The ACE2 gene expression was initially established in the heart, kidneys, and testes,^23^ while further studies showed a much broader distribution, such as the upper respiratory tract, lungs, intestine, liver, and pancreas.^24–26^ Moreover, neuropilin-1 (NRP1), expressed in the respiratory and olfactory epithelium, may be an additional cellular facilitator of SARS-CoV-2 cell entry and infectivity.^27^ In addition, an RNA sequencing analysis shows that although immune cells do not express ACE2 or TMPRSS2, another receptor for SARS-CoV-2, a transmembrane protein of the immunoglobulin cluster of differentiation (CD)147 provides a potential route for viral entry.^28,29^ SARS-CoV-2 can also exploit receptor-mediated endocytosis through interaction between its S protein with soluble ACE2 or soluble ACE2-vasopressin via angiotensin (Ang) II type receptor 1(AT1R) or arginine vasopressin receptor 1B (AVPR1B).^30^

After binding to the receptor, proteolytic cleavage of SARS-CoV-2 S protein enables the S2 subunit-assisted fusion of viral and cellular membranes. This process is mediated via certain host proteases including furin, cell surface transmembrane serine proteases 2 (TMPRSS2),^31^ cathepsins B and L, factor Xa and elastase. An insertion of four amino acids in the S1/S2 site of S protein provides a minimal cleavage motif (RRAR) recognized by proprotein convertase furin, which is a unique feature of SARS-CoV-2. S protein is cleaved at the S1/S2 site by furin and subsequent at the S2’ site by TMPRSS2, triggering an irreversible and extensive conformational change to mediate membrane fusion.^32–34^ Besides, inside the endosome, a pH-dependent endosomal protease cathepsin L can facilitate the cleavage and proteolytical activation of S protein for fusion within the endosomal membrane.^35^ Inhibition of these proteases, particularly TMPRSS2,^36^ might constitute a treatment option to treat COVID-19.

The following SARS-CoV-2 life cycle inside the cell is similar to that of other coronaviruses.^37^ SARS-CoV-2 releases viral genome into the cytoplasm to induce translation of open reading frame (ORF)1a and ORF1b into the large replicase polyproteins 1a (pp1a) and pp1ab. Subsequently, two viral proteases, a papain-like protease (PLpro) and a 3C-like protease (3CLpro) cleave pp1a and pp1ab into 16 nonstructural proteins (nsps) that assemble into replication-transcription complexes (RTCs) for RNA synthesis.^38^ The RNA-dependent RNA polymerase (RdRp) is the central enzyme of RTCs. The RTCs produce new genomic RNA by continuous synthesis and a set of subgenomic RNA.^33^ These further are translated into respective viral proteins. The viral structural proteins (S, E, and M) traffic through the endoplasmic reticulum (ER) to ER–Golgi intermediate compartment (ERGIC). The N protein package genomic RNA into helical structures in the cytoplasm, and interact with hydrophobic M protein in the ERGIC that serve to direct assembly and budding of the mature virion.^39^ These virions are transported to the cell surface in vesicles and then released through exocytosis into the extracellular region.^33,34^ The development of effective therapeutic strategies for COVID-19 relies on the knowledge of molecular mechanisms of SARS-CoV-2 infection.

#### Emerging SARS-CoV-2 variants

Like other RNA viruses, SARS-CoV-2 tends to evolve rapidly, producing mutants that differed significantly from its ancestral strains. A classification system was established to distinguish the emerging SARS-CoV-2 variants into variants of concern (VOCs) and variants of interest (VOIs). There are currently five main designated VOCs, including Alpha, Beta, Gamma, Delta, and Omicron variants. Alpha, Beta, Gamma, and Delta variants were first identified in the UK, South Africa, Brazil, and India, respectively.^40^ VOCs have been associated with increased transmissibility and viral virulence, decreased diagnostic sensitivity, and potential influence on vaccination.^41^ All VOCs carry mutation D614G that may enhance infectivity of SARS-CoV-2 by assembling more functional S protein into the virion.^42^ N501Y mutation located within the RBD is common to all variants except the Delta variant that contributes to increased affinity of the S protein to ACE2, promoting the viral attachment and its subsequent entry into the host cells.^43,44^

Alpha variant is also known as lineage B.1.1.7. Three B.1.1.7 S protein mutations are of particular concern: a two-amino-acid deletion at position 69–70 of the NTD; N501Y; and P681H, proximal to the furin cleavage site.^45^ Mutation P681H is a known region of importance for infection and transmission.^28,46^ The ∆H69/∆V70 deletion results in increased infectivity and evasion of the immune response.^20^ Beta variant known as multiple B.1.351 sublineages, includes nine mutations in S protein. K417N, E484K, and N501Y are located in the RBD.^19^ These changes confer enhanced affinity for ACE2^44^ and help to escape from neutralization and reduce neutralization sensitivity to convalescent plasma.^47^ Gamma variant, also known as lineage P.1, harbors ten mutations in the S protein. Three mutations (L18F, K417N, E484K) are located in the RBD.^18,48^ This variant may have reduced neutralization by monoclonal antibody therapies, convalescent sera, and postvaccination sera.^49^ Delta variant referred to as the B.1.617.2 lineage, has a highly mutated NTD (T19R, G142D, Δ156-157, R158G, A222V). According to the reports,^50,51^ the Delta variant was resistant to neutralization by some anti-NTD and anti-RBD monoclonal antibodies.^52^ The Delta Plus variant also known as B.1.617.2.1 or AY.1, is a sublineage of the Delta variant. Five key mutations (T95I, A222V, G142D, R158G, and K417N) were significantly more prevalent in the Delta Plus than in the Delta variant.^53^ On 26 November 2021, WHO designated the newly emerging variant B.1.1.529 a VOC, named Omicron, which has a total of 60 mutations.

### Indirect mechanisms of SARS-CoV-2 infection

#### Dysregulation of the immune response

**Fig. 1:**
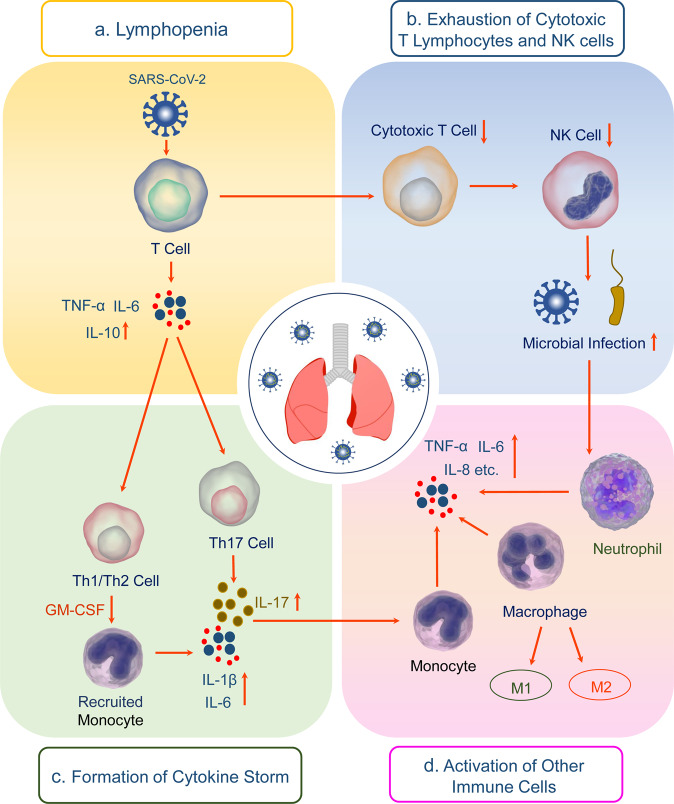
The potential mechanisms of SARS-CoV-2-induced immunopathology. **a** Lymphopenia, **b** Exhaustion of cytotoxic T lymphocytes and NK cells, **c** Cytokine storm, **d** Activation of other immune cells contribute to the pathogenesis and exacerbation of COVID-19

The pathogenesis of COVID-19 is triggered by SARS-CoV-2 infection and amplified by dysregulated immune responses. Impaired immune system and hyperinflammation induced by SARS-CoV-2, instead of the direct detrimental toxicity of virus, may account for severe disease with multiple organ involvement in severe and critically ill COVID-19 patients.^54^ Patients with ARDS and extrapulmonary complications have increased levels of circulating proinflammatory cytokines, chemokines and systemic inflammatory markers such as ferritin, lactate dehydrogenase (LDH), c-reactive protein (CRP), D-dimer, and neutrophil-to-lymphocyte ratio.^55^ As summarized in Fig. 1, increased proinflammatory of cytokines, lymphocytopenia, lymphocyte exhaustion, and upregulated antibodies may be involved in the immune pathogenesis of COVID-19.^15^

##### Innate immune response

As a frontline of defense, the innate immune response to SARS-CoV-2 infection triggers several signaling pathways to induce the production of IFN, proinflammatory cytokines and chemokines, and initiate adaptive immunity against SARS-CoV-2. Epithelial cells in the respiratory tract acting as the first line of innate immune sensing of SARS-CoV-2 infection, are a major source of chemokine interleukin (IL)-8 that plays an important role in regulating lung neutrophil recruitment and survival. Alveolar neutrophils and macrophages subsequently trigger the innate immune response to the virus.^56^ Neutrophils engulf and kill the viruses through the release of neutrophil extracellular traps (NETs), reactive oxygen species (ROS), and antimicrobial peptides.^57^ The enhanced infiltration of granulocytes and monocyte-macrophages is a common phenomenon in severe COVID-19 cases. Monocytes and macrophages are involved in the exacerbated and hypersensitive reactions contributing to the organ damage.^58^ Besides, multiple studies have shown decreased numbers and functionally exhaustion of natural killer (NK) cells during SARS-CoV-2 infection.^59^ The diminished NK cell cytotoxicity and immune regulation result in a critical inflammatory phenotype in COVID-19.^60^

The pattern-recognition receptors (PRRs) in/on the immune cells, involving toll-like receptors (TLRs) such as TLR3 or TLR7, and retinoic acid-inducible gene (RIG)-I-like receptors (RLRs) such as RIG-I and the melanoma differentiation-associated gene 5 (MDA5) recognize the pathogen-associated molecular patterns (PAMPs) derived from SARS-CoV-2, such as viral ssRNA genome, replication intermediates or double-stranded RNA (dsRNA), thereby initiating the antiviral responses.^61^ Endosomal TLR7 expressed in monocytes, dendritic cells (DCs) and macrophages recognizes viral genomic RNA and subsequently results in the activation of Janus kinase (JAK)/signal transducer and activator of transcription (STAT) signaling pathways, and its downstream transcription factors, activator protein-1 (AP-1), nuclear factor kappa B (NF-κB), interferon response factor (IRF) 3, and IRF7.^62^ These activated signaling pathways and transcription factors induce the rapid production of proinflammatory cytokines.^63^

The immune hallmark of severe COVID-19 is exaggerated secretion of cytokines, such as interleukin (IL)-1β, IL-2, IL-6, IL-8, IL-10, granulocyte macrophage-colony stimulating factor (GM-CSF), IFN-γ, and TNF-α, interferon-inducible protein-10 (IP10), macrophage inflammatory protein (MIP)-1α, tumor necrosis factor (TNF)-α, etc. This life-threatening condition related to systemic inflammation with sometimes lethal consequences is known as cytokine storm syndrome (CSS) or cytokine release syndrome (CRS), or just cytokine storm.^64,65^ Cytokine storm, an overwhelming inflammatory response results in the pathophysiology and mortality of SARS-CoV-2 infection. Cytokine storm is closely associated with macrophage activation syndrome (MAS), which is characterized by inflammatory systemic abnormality such as pancytopenia, hyperferritinemia, coagulopathy, hemodynamic instability, liver failure, neurological disorder, and can lead to ARDS or even multiorgan damage associated with unfavorable prognosis of COVID-19.^66^ MAS is resulted from the excessive proliferation of differentiated macrophages that cause hemophagocytosis and hypercytokinemia.^67,68^

As an important pleiotropic proinflammatory mediator, IL-6 is the main driver of cytokine storm through promoting the proliferation of myeloid progenitor cells and activation of leukocytes, inducing pyrexia, and escalating the secretion of acute-phase proteins in the severe cases of COVID-19.^69^ SARS-CoV-2 infection induces a wide range of immune cells including macrophages, neutrophils, DCs and lymphocytes to secrete excessive amounts of IL-6.^70,71^ Excessive IL-6 promotes the differentiation of Th17 cells and stimulates IL-17 production,^72^ and further recruits neutrophils, monocytes, and macrophages to the site of infection and inflammation and triggers a cascade of inflammatory cytokines, such as IL-1β and IL-6, leading to an IL-6 burst in its amplification cycle.^73^ Increased levels of IL-6 are significantly associated with the disease severity and adverse clinical outcome of COVID-19.^74^ The IL-6 signaling cascade is initiated by IL-6 binding to the membrane-bound or soluble IL-6 receptor (IL-6R) and a second transmembrane protein, glycoprotein 130 (gp130), which is referred to as classic signaling or trans-signaling, respectively.^75^ IL-6 classic signaling may have homeostatic and anti-inflammatory effects, whereas trans-signaling may regulate proinflammatory response.^76^ Expression of IL-6R is restricted to cells including hepatocytes and immune cells, but gp130 is ubiquitously expressed, possibly explaining the pleiotropic functions of IL-6. Recombinant humanized monoclonal antibodies against IL-6R or IL-6 are drug candidates for managing the cytokine storm secondary to SARS-CoV-2 infection^77^ through inhibiting the intercellular signaling pathway in gp130 expressing cells.

GM-CSF also has a critical role in mediating cytokine storm. Because of its function as a proinflammatory cytokine and a myeloid cell growth factor, GM-CSF may be another important driver of the immunopathological sequelae of SARS-CoV-2 infection.^69^ Upon SARS-CoV-2 infection, CD4^+^ T lymphocytes are rapidly differentiated into pathogenic T helper (Th) 1 cells that produce IL-6 and GM-CSF, subsequently inducing CD14^+^CD16^+^ monocytes to secrete high levels of IL-6 and GM-CSF and worsen the cytokine storm.^78^ Hence, a monoclonal antibody against GM-CSF may be effective to attenuate the immunopathogenesis of COVID-19.

IFN is innate cytokine that functions as the first-line defense against viral infection. Type I IFN, including IFN-α and IFN-β, triggers the expression of IFN-stimulated genes (ISGs), which directly suppress viral replication by various mechanisms, involving degradation of viral RNA or inhibition of viral transcription or translation.^79,80^ More than one-third of SARS-CoV proteins have inhibitory effects on type I IFN-mediated antiviral immune responses.^81^ Given most of the SARS-CoV-2 proteins exhibit high amino acid-sequence homology with those of SARS-CoV, it is speculated that SARS-CoV-2 proteins may exhibit inhibitory effects on IFN responses through similar mechanisms.^80^ SARS-CoV-2 have evolved mechanisms to evade the antiviral function of type I and III IFNs, including interference with the induction of IFN production or the downstream signaling pathways after IFN binding to the IFN receptors (IFNRs).^80^ Patients with severe or critically ill COVID-19 had highly impaired type I IFN response, characterized by low production and activity of type I IFN and ISGs.^82^ Compared to asymptomatic or mild COVID-19, severe cases are more likely to carry mutations in genes involved in type I IFN pathways or have autoantibodies against IFN that can neutralize high concentrations of type I IFN in vitro.^83,84^ However, increasing evidence also shows contradictory findings that severe COVID-19 patients have a robust type I IFN response, contrary to a delayed and likely suppressed IFN response found in the early phase of infection.^85^ Deeper understanding of the roles of IFNs response in SARS-CoV-2 infection is warrant further investigation.

##### Adaptive immune response

The adaptive immune system is also called specific or acquired immunity, including cellular immunity carried out by T cells and humoral immunity mediated by B cells that elicit protective immune response against pathogens in an antigen-specific manner.^86^ During viral infection, an effective adaptive immune response plays a crucial role in eliminating the virus and preventing the disease progression.^87^ Induction of an adaptive immune response against pathogens relies on the initial recognition and capture of antigens by antigen presenting cell (APC). The viral antigens are identified, processed, and presented by APCs to activate and guide the differentiation of CD4^+^ and CD8^+^ T cells into effector and memory cells.^88^ After being activated, CD4^+^ T cells differentiate into Th1, Th2 effector cells, and other subsets, characterized by distinct cytokine pattern.^89^ Th cells play critical roles in orchestrating the adaptive immune responses, through secretion of cytokines and chemokines that recruit immune cells and stimulate B cell differentiation and antibody production as well as activate CD8^+^ cytotoxic T lymphocytes (CTLs). Th1 cells produce IFN-γ, IL-2, and lymphotoxin α (LTα), and mediate immune responses against intracellular pathogens, whereas Th2 cells produce IL-4, IL-5, IL-9, IL-10, IL-13, and IL-25, and mediate host defense against extracellular parasites.^90^ CTLs can directly kill the virus-infected cells via exocytosis of lytic granules that contain perforin and granzymes or via the Fas pathway.^91^ T-follicular helper (Tfh) cells are a specialized subset of CD4^+^ T cells that can activate B cells to produce antibodies. The neutralizing antibodies exert protective activities through blocking SARS-CoV-2 infection in a later phase and conferring protection against future infection.^92^

Lymphopenia, particularly in peripheral CD4^+^ and CD8^+^ T cells, is frequently found and an early immunologic indicator of impending severe COVID-19.^93,94^ This lymphocytes depletion could be a manifestation of imbalance in both arms of immune responses, leading to dysregulated IFN production, hyperactivated neutrophils and macrophages, and delayed viral clearance. The prevalence of lymphopenia differed among the patients with different disease severities, with 72.7% developed in severe cases and 10.0% in the moderate case.^94^ Patients with severe COVID-19 showed considerably decrease in the counts of circulating memory CD4^+^ T cells, CD8^+^ T cells and regulatory T cells (Tregs).^94^ Despite reduced CD8^+^ T-cell counts, their histocompatibility complex (MHC) II cell surface receptor (HLA-DR) expression was higher in patients with severe COVID-19 than moderate cases. HLA-DR is primarily recognized as a marker of T-cell activation, but a recent study shows that CD8^+^HLA-DR^+^ T cells may constitute a Treg cell subset,^95^ and have immunosuppressive properties involving the inhibitory molecule the cytotoxic T lymphocyte antigen 4 (CTLA-4). High expressions of perforin and granzyme B in CD8^+^ T cells, low levels of TNF-α and IFN-γ in CD4^+^ T cells were related to disease severity of COVID-19.^96^ Moreover, CD8^+^ T cells more frequently displayed an exhausted phenotype in the severe COVID-19 cases. Patients with overtly symptomatic COVID-19 showed increased programmed cell death protein-1 (PD-1) and T-cell immunoglobulin domain and mucin domain-3 (TIM-3) expressions on CD8^+^T cells.^97^ These results indicate that functional impairment or exhaustion of T cells is correlated with disease severity and prognosis of patients with COVID-19. Moreover, SARS-CoV-2 infection may induce the downregulation of the MHC II expression on B cells, leading to decreased acquired immunity activation.^98^ An increased SARS-CoV-2-specific IgG antibody responses are strongly correlated with disease severity,^99^ suggesting that activation of B cells in severe COVID-19 patients is associated with adverse outcome.

Multiple underlying mechanisms may be responsible for lymphopenia and lymphocyte dysfunction. SARS-CoV-2 infects primarily epithelial cells in the respiratory tract through binding of S protein to ACE2. It is hypothesized that SARS-CoV-2 may suppress adaptive cellular immune response through infecting certain immune cells. However, some studies have demonstrated that only limited pulmonary macrophages or monocytes may express ACE2,^100^ which raises the possibility of the presence of additional receptors or cellular entry route such as antibody-dependent enhancement (ADE), granting SARS-CoV-2 an opportunity to infect host immune cells. The reduced T-cell numbers were inversely associated with IL-6, IL-10, and TNF-α levels. This phenomenon indicates that increased production of inflammatory cytokines may promote T-cell exhaustion and apoptosis that accompanies disease progression.^101^ Soluble IL-2 receptor can negatively regulate CD8^+^ T cells and induce lymphopenia via inhibition of IL-2 signaling.^102^ Moreover, lymphoid organ atrophy, such as the spleen and lymph node leads to further impairments of lymphocyte.^103^ Severe COVID-19 patients had an elevated level of lactic acid in the blood, which can suppress the proliferation of lymphocytes.^104^ Neutrophils with suppressive properties such as granulocytic myeloid-derived suppressor cells (G-MDSCs) and their possible role in suppressing CD4^+^ and CD8^+^ T lymphocytes expansion may also give rise to lymphopenia in severe SARS-CoV-2 infection.^105^

#### Endothelial cell damage

Endothelial biomarkers including von Willebrand factor (vWF), soluble P-selectin, and soluble thromboregulatory protein were elevated in severe COVID-19 patients, highlighting the importance of endothelial injury in the pathogenesis of COVID-19.^106^ Excessive matrix metalloproteinase-1 (MMP-1) and endothelial cell overactivation as evidenced by elevated soluble CD146, vascular cell adhesion molecule-1 (VCAM-1) and intercellular adhesion molecule-1 (ICAM-1) are associated with disease severity of patients with COVID-19.^107^ In addition, Ang II, soluble E-selectin (sE-sel), and soluble thrombomodulin were elevated only in critically ill patients, while only vWF antigen increased with disease severity. Therefore, as markers of endothelial injury, circulating vWF and high molecular weight multimers are the best predictors of in-hospital mortality.^108^

Patients with COVID-19 have severe endothelial damage in their lungs, including viral invasion and rupture of the endothelial cell membrane.^109^ Another study identified the co-presence of SARS-CoV-2 N protein and ACE2 receptor on the pulmonary vascular endothelium in postmortem COVID-19 patient samples.^110^ Moreover, IFN-α or -β can promote SARS-CoV-2 pulmonary vascular infection by inducing the expression of ACE2 in human primary lung endothelial cells.^111^ S1 and S2 subunits of S protein mediate attachment and membrane fusion, respectively. In primary human pulmonary microvascular endothelial cells that naturally express ACE2, S1 subunits instead of intact S protein reduces transendothelial resistance (TER) and barrier function.^112^ Plasma mediators of severe COVID-19 patients can cause lung endothelial barrier failure.^113^

SARS-CoV-2 can not only cause lung endothelial cell damage, but also affect the endothelial cells in extrapulmonary organs. The study found that the endothelial cells of the vascular bed of different organs are affected in patients with COVID-19.^114^ Besides the lungs, endothelial-related inflammatory cells and apoptotic bodies clusters were found in the heart and small intestine. Moreover, another patient with COVID-19 has also found obvious endotheliitis in the heart, liver, kidney, and small intestine. In the circulatory system, COVID-19-induced endodermatitis is a small vasculitis and does not involve the major coronary arteries.^115^ Renal biopsy also revealed endothelial abnormalities, ranging from mild injury with enlarged subcutaneous space and/or loss of endothelial cell windows in the glomeruli to severe injury with swollen endothelial cells in the glomerular portal arterioles and fibrin thrombus.^116^

**Fig. 2:**
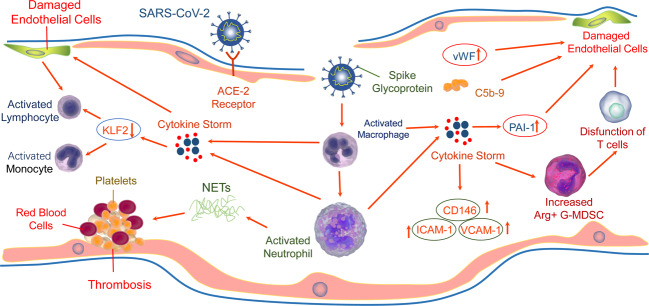
Mechanisms of SARS-CoV-2 induced to endotheliopathy in COVID-19. SARS-CoV-2 directly invades endothelial cells or indirectly induces cytokine storm to cause endothelial cell damage. On the one hand, the SARS-CoV-2 receptor ACE2 expressed on the surface of endothelial cells can be directly invaded by the virus. On the other hand, cytokine storm destroys endothelial cells by inducing the release of PAI-1, promoting the degradation of endothelial glycocalyx to release HA fragments and destroying the endothelial barrier; downregulating the expression of KLF2 to induce adhesion and infiltration of monocytes/macrophages, or by immune dysregulation such as increased NETS generation and T-cell dysfunction. Finally, endothelial dysfunction could be further aggravated by complement activation, thrombosis, coagulation disorders and activation of immune cells. Meanwhile, circulating endothelial injury markers including vWF and sCD146 were elevated

Endothelial injury may occur through direct invasion of endothelial cells or indirect effect of SARS-CoV-2 (Fig. 2). ACE2 on the surface of endothelial cells can be invaded by SARS-CoV-2.^117^ SARS-CoV-2 can also infect the endothelial cells of extrapulmonary organs. ACE2 was present in arterial and venous endothelial cells of all studied organs.^24^ The structure of the virus inclusion body was found in the kidney endothelial cells of patients who died from COVID-19 through electron microscopy.^114^ In addition, SARS-CoV-2 have been found in neural and capillary endothelial cells of frontal lobe in COVID-19 patients.^118^ The S protein of SARS-CoV-2 can directly damage endothelial cells, manifested as impaired mitochondrial function and endothelial nitric oxide synthase (eNOS) activity, as well as downregulation of ACE2, which may further aggravate endothelial dysfunction due to the disorders of RAS.^119^

In addition to directly infecting endothelial cells, SARS-CoV-2-related cytokine storm and invasive inflammation contribute to endothelial damage in extrapulmonary organs.^114,120^ The plasma of patients with severe COVID-19 can induce endothelial damage.^113,121^ The excessive inflammatory effect of cytokine storm may lead to endothelial activation and dysfunction. High serum TNF-α and IL-1β levels in patients with COVID-19 may downregulate the Kruppel-like factor 2 (KLF2) expression in human endothelial cells, and subsequently induce monocyte adhesion, leading to endodermatitis characterized by endothelial dysfunction and hypercoagulability, and lymphocytic monocyte infiltration in patients with COVID-19.^122^ IL-6 trans-signaling mediates the plasminogen activator inhibitor-1 (PAI-1) releasing from vascular endothelial cells in CRS. Increased levels of PAI-1 can result in endothelial dysfunction, induce cell senescence, thereby promoting local hypoxia.^123^ In the liver sinusoidal endothelial cells (LSEC), IL-6 trans-signaling leads to proinflammatory and procoagulant states endothelial lesions, and liver injury in COVID-19.^124^ In addition, macrophage and complement activation^125^ play a crucial role in endothelial damage and thrombosis in SARS-CoV-2 infection.^126^ Hyaluronic acid (HA) is a ubiquitous glycosaminoglycan and main constituent of the glycocalyx that is anchored to the vascular lumen and regulates a diverse repertoire of endothelial functions. SARS-CoV-2 infection-induced cytokine storm leads to abnormal degradation of endodermis glycocalyx, resulting in HA fragments that may cause dysfunction of endothelial barrier and vascular hyperpermeability in a ROCK- and CD44-dependent manner.^127,128^ The circulating granulocyte-myeloid-derived suppressor cells (G-MDSC) expressing high levels of arginase-1(Arg1) increased significantly in COVID-19 patients, which can deplete arginine in the plasma and inhibit T-cell receptor signal transduction, thereby leading to T-cell dysfunction, also impairing the production of nitric oxide and increasing endothelial cell dysfunction, and promoting intravascular coagulation.^129^ Moreover, due to sustained immune activation during COVID-19 convalescence, activated and infected endothelial cells may be susceptible to direct T-cell-mediated cytotoxicity that may intensify endothelial dysfunction in patients with COVID-19.^130^

Endothelial injury in COVID-19 patients can lead to dysregulation of coagulation factors and complement, as well as excessive activation of platelets, resulting in thrombosis and eventually clotting disorders. Moreover, endothelial injury recruits and activates immune cells including neutrophils and macrophages, as well as promotes the release of cytokines and the formation of NETs, etc., leading to proinflammatory reactions, which may further aggravate endothelial injury.

#### Coagulopathy

Coagulopathy is another common feature of COVID-19, which is depicted with thrombocytopenia, prolonged prothrombin time (PT), increased D-dimer levels, and/or decreased fibrinogen levels. In COVID-19, there were elevated D-dimer levels and fibrin degradation products accompanied by mild to moderate increase in PT and activated partial thromboplastin times (APTT).^7^ About 60% ICU patients had abnormally elevated D-dimer levels compared with a prevalence of 43% in non-severe patients.^131^ Moreover, increased D-dimer levels were associated with adverse prognosis.^132^ In severe COVID-19 patients, thrombotic complications are common due to the prothrombotic state and contribute significantly to mortality and morbidity.

The hypercoagulable state is more frequent in elderly COVID‐19 patients.^133^ COVID-19 patients with hypertension or diabetes mellitus are more likely to suffer lower extremity complications,^134^ and coagulopathy is a major extrapulmonary risk factor for mortality in hospitalized COVID-19 with type 2 diabetes rather than acute kidney injury (AKI) and acute cardiac injury.^135^

The most common thrombotic complications include deep vein thrombosis (DVT), pulmonary embolism (PE), and DIC. In severe COVID-19 patients admitted to ICU, the frequency of thrombotic complications was 31% of 184, composed by 27% Venous thromboembolism (VTE) events and 3.7% arterial thrombotic events. Moreover, age and coagulopathy were independent predictors of thrombotic complications.^136^ In total, 32 (24%) cases of PE were identified with computed tomography pulmonary angiogram (CTPA) in 135 COVID-19 patients, and the rate increased to 50% in ICU patients.^137^ Existing data of autopsies from COVID-19 patients showed that massive PE accounted for one-third of causes of death, with an additional one fourth with recent DVT but without PE. Overall, 75% of them were male and two-thirds were noted to have recent thrombosis in prostatic venous plexus.^138^ In all, 8% of patients matched overt DIC according to the International Society on Thrombosis and Hemostasis diagnostic criteria (ISTH).^7^ DIC was developed in 71.4% of patients who died from COVID-19, while it only occurred in 0.6% of those who survived.^7^

SARS-CoV-2-induced excessive immune response and inflammatory injury lead to endothelial dysfunction, dysregulation of coagulation factors and complement, platelet activation and death, as well as release of NETs, thereby promoting thrombosis (Fig. 2), and eventually resulting in an imbalance of the coagulation system, coagulation dysfunction, and a range of pulmonary and extrapulmonary complications. These multiple factors eventually result in pathological angiogenesis, thrombosis, and clotting disorders.

Viral infection can lead to systemic hypoxia, which may cause coagulation protein imbalance and increased activation of the coagulation cascade.^139^ Meanwhile, proteomics showed that in deceased COVID-19 patients, several coagulation factors such as prothrombin (F2), factor XI, XII, and XIIIa, etc. involved in the coagulation, anticoagulation, and fibrinolysis systems, were dysregulated, which may lead to coagulopathy in COVID-19.^140^ Elevated plasma levels of complement component 5 (C5) activation products, C5a and C5b-9 in the patients with COVID-19 indicated complement activation.^141^ S protein of SARS-CoV-2 can interfere with the function of complement factor H to activate complement bypass, and directly block the combination of complement factor H with heparin, leading to complement imbalance.^142^

In the context of COVID-19, platelets and platelet activation biomarkers are elevated in deceased patients.^143,144^ SARS-CoV-2 binds to platelets through S protein to promote platelet activation,^145^ activated platelets drive monocytes aggregation and increase the tissue factor (TF) expression, ultimately leading to the deterioration of coagulation.^146^ Transcriptomic analysis showed that SARS-CoV-2 infection markedly altered expression of genes related to platelet and triggered strong platelet hyperreactivity, leading to increased platelet activation and aggregation by activating mitogen-activated protein kinase (MAPK) pathway and subsequent thrombin production.^147^ SARS-CoV-2 particles were internalized by platelets in an ACE2-independent manner, resulting in rapid digestion, programmed cell death, and release of extracellular vesicles.^148^

NETs are a key factor for COVID-19-associated immunothrombosis, and plasma of patients with COVID-19 can induce the formation of NETs.^149^ Pulmonary autopsy also confirmed infiltration of NETs.^150^ Overactivated platelets recruit neutrophils, which increase the release of NETs.^151^ In addition, SARS-CoV-2 triggered NETs dependent of ACE2, viral replication, serine proteases, and protein arginine deiminase 4 (PAD4).^152^ NETs bind to the factor XII zymogen and induce coagulation in a factor XII-dependent manner.^153^ The accumulation of NETs in the vessels results in rapid occlusion of the affected vessels, microcirculation disruption, and organ injury.^154^

Dysregulation of immune thrombosis is a key indicator of the disease severity of COVID-19.^151^ Endothelial cell injury and activation, thrombin activation, platelet activation and aggregation, as well as neutrophil recruitment and activation are involved in the complex processes of immunothrombosis. In addition, COVID-19 patients showed excessive activation of non-phagocytic cell oxidase (Nox) 2, which induced oxidative stress to cause vascular occlusion, platelet aggregation, and ultimately thrombosis.^155^

#### Dysregulation of RAS system

Apart from acting as an entry receptor for SARS-CoV-2, ACE2 seems to be a protective molecule for the heart and kidneys, and viral binding may deregulate its protective effect. RAS system is involved in the regulation of cardiac, renal, and vascular physiology.^13^ RAS dysfunction is related to the development of acute lung injury and ARDS, and associated with poor prognosis.^156^ ACE2 negatively regulates RAS system and maintains physiological homeostasis, by converting Ang I to the nonapeptide Ang 1–9, an inactive form of Ang, and Ang II to the counter‑regulatory heptapeptide, Ang 1–7.^157^ These peptides have vasodilatory and antiproliferative effects, and have protective functions by interacting with MAS1 receptor, which is a G protein-coupled receptor.^158^ As a potent vasoconstrictor, Ang II mediates vasoconstriction via AT1R and vasodilatation through Ang II type 2 receptor (AT2R). In the context of SARS-CoV-2 infection, cleavage of ACE2 by a disintegrin and metallopeptidase domain 17 (ADAM17) and TMPRSS2 facilitates cell entry.^158^ This process may lead to ACE2 shedding and loss of protective function of ACE2, subsequently increase Ang II levels and finally induce AT1R stimulation and AT2R inactivation.^159^ This process triggers the secretion of aldosterone, vasopressin, and adrenocorticotropic hormone (ACTH), hypokalemia, sodium reabsorption, inflammation, cell proliferation, and lung injury. ACE2/Ang 1–7/MAS axis counterbalances the deleterious effect of the ACE/Ang II/AT1R axis. ACE2 downregulation leads to pulmonary vascular hyperpermeability and coagulation, which in turn results in multiple organ damage.^160^ The ACE2 downregulation promotes pathological changes in acute lung injury and participates in inflammatory and fibrotic responses,^14,161^ and aggravates disease progression of COVID-19.^162^ ACE2 deficiency in patients with advanced age, comorbidities such as cardiovascular disease, diabetes mellitus, or increased shedding of ACE2 due to the infection, may result in overactivity of the ACE/Ang II/AT1R axis, contributing to enhanced inflammation and thrombosis.^163^ Therefore, ACE2 acts as a key mediator and a therapeutic target for COVID-19.

## ARDS and its association with extrapulmonary complications

SARS-CoV-2 predominantly displays a respiratory tissue tropism and commonly causes pulmonary complications such as pneumonia and, in severe cases, ARDS or hypoxemic respiratory failure. Meta-analysis has shown that 18% of patients hospitalized with COVID-19 had severe disease, with 15% developing ARDS.^164^ COVID‐19 associated ARDS is more likely to have worse outcomes than ARDS secondary to other predisposing causes, with mortality ranging from 26 to 61.5% in patients admitted to intensive critical care unit (ICU) and from 65.7 to 94% in those receiving mechanical ventilation.^165^

Although SARS-CoV-2 can affect various tissues and organs through widely distributed ACE2 in cardiovascular, renal, and gastrointestinal systems, etc. During the initial phases of infection, SARS-CoV-2 may be restricted to the respiratory tract, thus currently the laboratory diagnosis of SARS-CoV-2 infection is based on the detection of viral nucleic acid in the nasopharyngeal (NP) or oropharingeal (OP) swab. The intense intracellular replication of SARS-CoV-2 causes programmed cell death including apoptosis and pyroptosis induced by inflammasome, resulting in capillary leakage and proinflammatory cytokines release and tissue damage.^166^ The activation of inflammasome is triggered by viroporins-induced ion influx or by endoplasmic reticulum stress response. Pyroptosis of infected airway endothelial cells may allow SARS-CoV-2 to leak out into the bloodstream and circulate to other organs and infect ACE2-expressing cells at local sites, resulting in extrapulmonary organ injuries.^114^

Airway epithelial cells are the first gateway for SARS-CoV-2 invasion. Initial infection site is the ciliated cells within proximal airway epithelium, but in severe cases, infection or injures induced by SARS-CoV-2 occurs diffusely in the alveolar epithelium, leading to gas-exchange impairment and respiratory failure with a high mortality rate. In the gas-exchange region of the distal lung, the alveolar facultative progenitors, alveolar type 2 (AT2) epithelial cells are the main target of infection.^167^ AT2 cells are specialized to synthesize and secrete surfactant, which is indispensable to reduce alveolar surface tension and prevent alveoli from collapsing and is involved in pulmonary host defense. Infection in this region induces progressive hypoxia and inflammatory cell infiltrates, which drive ARDS in severe cases of COVID-19.^168^ AT2 cells also play a critical role in regulating alveolar hypercoagulation and fibrinolysis inhibition by PAI-1 and urokinase production. Infection of AT2 cells initiates the innate immune response that favors virus propagation to adjacent alveoli and perpetuates a hyperinflammatory state, resulting in ARDS with diffuse alveolar damage (DAD), microvasculature injury, hyaline membranes, thrombosis, and fibrin deposition in the alveoli.^169,170^

The evolution of ARDS can be divided into three phases, including acute exudative, proliferative, and fibrotic phases. In exudative phase, DAD and endothelial injury induce the formation of intra-alveolar hyaline membrane, as well as widening and edema in the lung interstitium. In the proliferative and fibrotic phases, AT2 cells hyperplasia, fibroblasts proliferation and chronic inflammation may lead to pulmonary fibrosis. Pulmonary fibrous strips and fibrosis were reported in 17% of COVID-19 patients.^171^ The hallmark in the pathophysiology of ARDS is the increase in permeability of the alveolar-capillary epithelial barrier that allows protein-rich fluid to enter the alveoli leading to pulmonary edema, hypoxemia, and consequent release of proinflammatory cytokines such as IL-1β, IL-6, IL-8, and TNF-α.^172^ Similar pathological changes of DAD in the lung are identified in COVID‐19 associated ARDS and the typical ARDS.^173^

Alveolar macrophages are critical for pathogen recognition, normal tissue homeostasis, the orchestration of lung inflammation and resolution of ARDS.^174^ Upon stimulation, alveolar macrophages can recruit neutrophils and monocytes via several chemokines such as IL-8 to the injury site in the lung. These cells contribute to the production of inflammatory mediators, such as ROS, proteases, cytokines, etc., which subsequently induce distal cell death, specifically AT2 epithelial cells. Moreover, alveolar macrophages can interact with lymphocytes, epithelial cells and mesenchymal stem cells (MSCs) in a paracrine manner, thereby augmenting inflammatory response and accentuating tissue injury.

ARDS is a progressive systemic inflammatory syndrome with lung involvement and extrapulmonary multi-organs damage. Elevated proinflammatory cytokines were observed in both bronchoalveolar lavage fluid (BALF) and plasma from patients with ARDS.^175^ COVID-19 associated ARDS is a typical “pulmonary” ARDS. The hallmark of severe ARDS secondary to COVID-19 is cytokine storm resulted from dysregulated inflammatory responses.^176^ In the meantime, the spillover of proinflammatory mediators into the peripheral bloodstream can maintain and augment the inflammatory response, causing extensive tissue damage to other organs. Endothelial cells are involved in the pathogeneses of both ARDS and extrapulmonary organ dysfunction, possibly through mediating systemic endotheliitis with marked infiltration of inflammatory cells and apoptotic bodies in various tissues and organs.^114^ The widespread endothelial inflammation alters integrity of vessel barrier and promotes procoagulant state and contributes to the tissue edema and organ ischemia, leading to histopathologic alterations and systemic complications in severe COVID-19 patients.^177^

Current evidence suggests that COVID-19-associated extrapulmonary organ injury can also be explained by cross-talk between the organs.^178^ Pulmonary complication is a key driver of increased mortality in patients with AKI, highlighting a bidirectional relationship. Recent studies confirmed the close relationship between alveolar and tubular damage, the lung–kidney cross-talk in ARDS.^179^ Cytokine such as IL-6 overproduction is involved in lung–kidney bidirectional damage.^180^ ARDS can induce renal medullary hypoxia, which is an additional insult to tubular cells.^180^ In addition, lung–heart,^181^ gut–lung,^182^ and brain–lung interactions,^183^ etc., have also been proposed as potential underlying mechanisms of SARS-CoV-2-induced multiorgan dysfunction.

## Extrapulmonary complications

**Fig. 3:**
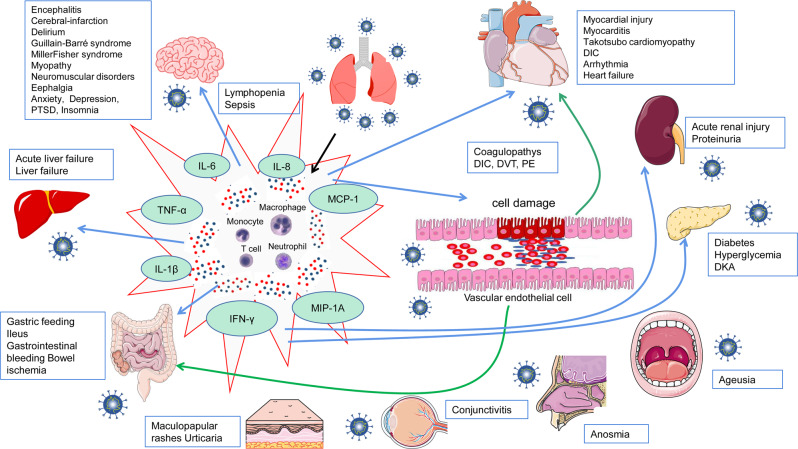
The extrapulmonary complications of COVID-19. SARS-CoV-2 infection has resulted in not only a pulmonary disease but also potentially systematic disease, which may cause long-term multiple organ-system complications including hyperinflammatory syndrome, vascular thrombosis, coagulopathy, cardiovascular, hepatobiliary, gastrointestinal, renal, neurologic, endocrinologic, ophthalmologic, nasal, oral, and dermatologic systems. Proposed mechanisms of the involvement of different organs or systems for COVID-19 caused by infection with SARS-CoV-2 include: direct viral toxicity through interaction of SARS-CoV-2 spike protein with the entry receptor ACE2; dysregulation of the immune response, T-cell lymphodepletion and hyperinflammation; endothelial cell damage and thromboinflammation. COVID-19 coronavirus disease 2019, SARS-CoV-2 severe acute respiratory syndrome coronavirus 2, DIC disseminated intravascular coagulation, PE pulmonary, DVT deep venous thrombosis, DKA diabetic ketoacidosis, PTSD post-traumatic stress disorder

In addition to the respiratory system, many other important organ systems are also vulnerable to the SARS-CoV-2 infection, resulting in several extrapulmonary manifestations and complications (Fig. 3). The systemic manifestations of COVID-19 vary, but these complications are largely interwoven by certain shared mechanisms, involving direct viral cytotoxicity, immune disturbances, endothelial damage and thromboinflammation, and ACE2-associated RAS system dysregulation.

### Cardiovascular complications

#### Adverse cardiovascular events of COVID-19

Cardiovascular system is frequently involved during the development and exacerbation of COVID-19, particularly in patients with preexisting cardiovascular diseases such as hypertension, heart failure or coronary heart disease. There are several potential mechanisms, involving myocardial injury, exacerbation of the underlying cardiovascular comorbidities, as well as cardiovascular adverse effects of the drugs used in the treatment of COVID-19.

Myocardial injury defined as elevated serum cardiac troponin I concentrations or abnormalities in electrocardiogram (ECG) or echocardiogram, is a common complication in the development and exacerbation of COVID-19. The incidence of myocardial injury differed among patients with different severities of COVID-19, with 10% in mild cases, roughly 30% in hospitalized patients on admission and ~50% during hospitalization.^184^ An early study of 138 patients hospitalized with COVID-19 in Wuhan showed that myocardial injury was observed in 7.2% of hospitalized COVID-19 patients and 22% of those in the ICU.^185^ A report from China showed that during hospitalization roughly 12% of patients without a history of cardiovascular diseases showed elevated levels of troponin or cardiac arrest. It is worth noting that elevated high-sensitivity troponin I was found in 46% of the deceased COVID-19 patients but only 1% of the survivors.^186^ COVID-19-related myocarditis is characterized by myocardial injury without an ischemic cause and inflammatory infiltrates.^187,188^ Acute and delayed-onset myocarditis have been reported in previous cohorts as well as the autopsy studies of COVID-19 deaths.^189^ Fulminant myocarditis and cardiogenic shock were accompanied by atrial and ventricular arrhythmias.^190^ Takotsubo cardiomyopathy is a non-ischemic cardiomyopathy characterized by transient weakening of the cardiomyocytes and subsequent ballooning of the apex.^191,192^ In all, 2–7.75% of COVID-19 patients presenting with acute coronary syndrome were diagnosed with stress-induced cardiomyopathy. Nearly one-third of the COVID-19 patients with myocardial involvement were complicated by cardiogenic shock.^191^ COVID-19 may predispose patients to arterial and venous thrombosis.^193^ The critically ill patients with COVID-19 have high venous thromboembolism risk of 31–40%.^136^ The incidence of disseminated intravascular coagulation (DIC) was 71.4% in COVID-19 deaths. Lung microvasculature fibrin deposition can result in ARDS in patients concomitantly diagnosed with DIC.^194^ The COVID-19 associated myocardial injury and subsequent cardiac dysfunction may cause cardiac arrhythmias. Relative tachycardia and bradycardia frequently occurred in mild to critically ill patients with COVID-19.^195^ In all, 16.7% of patients hospitalized with COVID-19 and 44% of those referred to ICU developed cardiac arrhythmia.^192,196,197^ Abnormal PR interval behavior with increasing heart rate and QT prolongation are frequently observed in critically ill patients.^196,198^ it remains unclear whether high prevalence of heart failure in patients hospitalized with COVID-19 with a known history of cardiac disease, results from worsening of preexisting left ventricular dysfunction or newly developed cardiomyopathy. An early report on 113 COVID-19 deaths showed high incidence of cardiac complications including heart failure and acute cardiac injury.^93^ Cardiogenic shock was developed in one-third of COVID-19 cases with myocardial involvement and carried a high mortality of 26%.^199^

Newborns and children are expected to be less susceptible to COVID-19 partly because of the reduced function of ACE2 receptors. SARS-CoV-2 infection appears to be asymptomatic or mild in most children, some may develop a severe inflammatory syndrome with symptoms similar to Kawasaki disease or toxic shock syndrome. This Kawasaki-like illness have been called the multisystem inflammatory syndrome in children (MIS-C).^200^ Of recovered 99 competitive athletes with asymptomatic or mild SARS-CoV-2 infection, 3.3% had myopericarditis or pericarditis, which is associated with exercise-induced ventricular arrhythmias or cardiac symptoms.^201^ Myocardial injury and left ventricular dysfunction in pregnant women had a high mortality rate of 13.3%, which was attributed to malignant arrhythmias.^202^

#### Pathogenesis of cardiovascular complications of COVID-19

COVID-19 related myocardial injury is frequently observed and is associated with poor prognosis. The central pathophysiology of COVID-19 related myocardial injury involves a complex interplay between viral tropism, dysregulated host immune response, alteration in ACE2 and RAS system homeostasis, the vascular dysfunction, myocardial oxygen supply–demand imbalance as well as microvascular and macrovascular thrombosis.^5,203,204^

The cardiovascular pathology of COVID-19 can result from a direct SARS-CoV-2 cardiotoxicity. Human pluripotent stem cell-derived cardiomyocytes (hPSC-CMs) expressing ACE2 are permissive to SARS-CoV-2 replication. Notably, SARS-CoV-2-infected hPSC-CMs exhibit progressively impaired contractile and electrophysiological properties, and extensive cell death.^205,206^ Cardiac stromal cells can be infected by SARS-CoV-2, which could contribute to myocardial injury. Moreover, stromal cells exposed to SARS-CoV-2 can evolve into hyperinflammatory and pro-fibrotic phenotypes via ACE2-independent mechanism.

Platelet activation plays an important role in the pathogenesis of thrombotic events and cardiovascular complications. S protein of SARS-CoV-2 induces platelet activation directly to facilitate leukocyte–platelet aggregate formation, the release of coagulation factors and inflammatory mediator, thereby resulting in thrombosis formation. Furthermore, the MAPK cascade, considered as a downstream signaling of ACE/Ang II, mediates the activation effect of SARS-CoV-2 on platelet.^145^

Abundant expression of Th1 and Th2 cytokines lead to direct cardiac immunological injury and chemotaxis of neutrophil and macrophage.^207,208^ The inflammasome activation in the patients with COVID-19 is strongly related to hypercoagulopathy and cytokine storm, contributing to the COVID-19-associated cardiac injury. Under certain cardiovascular conditions, the inflammatory response triggered by the NOD-like receptor family pyrin domain containing 3 (NLRP3) inflammasome activation leads to hyperinflammation, which promotes cardiac injury and could be targeted for the treatment of COVID-19.^209^

The damage-associated molecular patterns (DAMP) ligands of the receptor for advanced glycation end products (RAGE) may exacerbate the local responses to infection in the heart, leading to severe cell stress and death, which in turn result in endothelial dysfunction, immune cell activation, oxidative stress, and upregulation of distinct factors such as early growth response 1 (EGR1). The inexorable accumulation of advanced glycation end products (AGEs) and other DAMP RAGE ligands relevant to cardiometabolic perturbation may prime the organs for amplification of inflammatory and tissue-damaging mechanisms upon SARS-CoV-2 infection.^210^ Nox2 is upregulated in pneumonia and closely associated with troponin elevation. Nox2-derived oxidative stress may contribute to myocardial injury via production of ROS, and thus inhibition of Nox2 may have therapeutic potential for COVID-19.^211^ Alteration in RAS after SARS-CoV-2 infection could predispose bradykinin storm. Given that bradykinin and its metabolites are inducers of endothelium-dependent vasodilation, vascular permeability, and pain via the activation of the G protein-coupled receptors B1 and B2, this signaling could be a new therapeutic target of cardiovascular dysfunction and thromboembolism induced by COVID-19.^212^

### Renal complication

#### Adverse renal events of COVID-19

AKI is a frequent complication in inpatients with COVID-19, with an incidence ranging from 10 to 80%.^213–217^ A meta-analytic study including 49,692 COVID-19 patients demonstrated that AKI was a common and serious complication of COVID-19. The in-hospital mortality risk was significantly increased in COVID-19 patients complicated by AKI.^218^ Elevated serum creatinine and proteinuria are the main clinical features of COVID-19 with kidney injury. Another meta-analytic study^219^ including 4963 COVID-19 patients showed that 9.6% of patients had elevation of serum creatinine. Of these patients, 57.2% had proteinuria. Proteinuria was reported in COVID-19 patients who did not develop AKI, which may indicate subclinical renal damage. Proteinuria occurs in patients with nephropathy, and significant heterogeneity exists between studies.^220–222^

#### Pathogenesis of renal complications of COVID-19

Multiple possible mechanisms may be involved in COVID-19 associated AKI, including SARS-CoV-2-mediated injury, inflammatory response, cytokine storm SARS-CoV-2-induced, activation of the ACE/Ang II pathway, dysregulation of complement, hypercoagulation, and microangiopathy.^223^ In an autopsy study of 63 COVID-19 patients, the viral RNA presence in the kidneys is correlated with older age and increased comorbidities, as well as reduced survival time. These results indicate a potential association between the renal tropism of SARS-CoV-2 and adverse clinical outcome.^224^ Renal tubular epithelial cells and podocytes express ACE2,^225^ while the distal nephrons but not the proximal tubules express TMPRSS2.^223^

The hyperinflammatory state of COVID-19 can result in kidney injury. Previous studies have found that high levels of cytokine release and inflammatory response lead to microvascular dysfunction, capillary hyperpermeability and insufficient perfusion, causing renal microcirculatory dysfunction.^226^ The critically ill COVID-19 patient had increased IL-6 levels that were associated with kidney damage possibly due to lung–kidney cross-talk.^180^ The bidirectional relationship between alveolar and tubular damage, lung–kidney cross-talk in ARDS is confirmed by recent studies.^179^ ARDS can induce hypoxia in the renal medulla, which may result in renal tubular epithelial cells injury, subsequently leading to the upregulation of IL-6.^180^ Glomerular diseases have been found in COVID-19 patients with kidney involvement.^227^ The most common pathological feature of glomerular disease is collapsing glomerulopathy.^228–232^ Collapsing glomerulopathy is a distinct pathology related to COVID-19, which may affect patients carrying high-risk APOL1 genotypes.^233,234^ Kidney biopsy of COVID-19 patients who had APOL1 high-risk genotype showed collapsing glomerulopathy, tubuloreticular inclusions in endothelial cells, and acute tubular injury, without evidence of SARS-CoV-2 infection or replication in kidney cells.^227^ During viral infection, IFN and TLR3 activation is sufficient to upregulate APOL1 gene expression.^235^ These findings suggest plausible mechanisms involving “two-hit” of cytokine-mediated host response to SARS-CoV-2 infection and genetic susceptibility.^227,233^

Kidney injury may be related to blood coagulation disorder in COVID-19 patients. In a kidney autopsy report of a patient who died from COVID-19,^236^ the renal parenchyma showed diffuse coagulative cortical necrosis, with widespread glomerular microthrombi. Electron microscopy showed extensive cross-linked fibrin deposition and partially shed capillaries in the capillary lumen. It is suggested that thrombotic microangiopathy instead of DIC is manifestation of coagulopathy in COVID-19 patients with kidney injury.^237–239^ Glomerular ischemia and endothelial cell damage also appear in some cases.^240^ Glomerular ischemia was observed in patients with fibrin thrombi in the glomerular capillary loops, which may be related to coagulation activation in COVID-19 patients.^6,241^ In addition, an interaction between Ang II overactivity, and complement pathways could also influence AKI severity and outcomes.

COVID-19 patients often present the respiratory and gastrointestinal symptoms, which may cause fluid loss. Once the fluid is not refilled in time or insufficient, it may lead to insufficient renal perfusion. In a retrospective study of 5,449 COVID-19 patients,^216^ AKI occurred in 36.6% of patients, and a majority of AKI patients had urine sodium lower than 35 mmol/L, indicating a state of pre-renal azotemia. In addition, ARDS or respiratory failure can reduce cardiac output through hemodynamic changes and high chest pressure, which may cause systemic inflammation and reduced renal perfusion induced by hypoxemia, leading to AKI.^8^

Drug-induced nephrotoxicity may contributor to COVID-19-related kidney injury.^242^ Some antivirals, antibiotics, and nonsteroidal anti-inflammatory drugs (NSAIDs) given to patients with COVID-19 during hospitalization may have possible nephrotoxicity and be involved in the development of AKI.^243^ A retrospective observational study showed that exposure to vancomycin and use of NSAIDs were risk factors associated with the development of AKI.^244^

### Gastrointestinal complications

#### Adverse gastrointestinal events of COVID-19

Diarrhea and other gastrointestinal symptoms are frequent in COVID-19 patients.^1,93,245^ Severe COVID-19 patients are more likely to develop gastrointestinal symptoms. The presence of digestive symptoms is associated with the disease severity.^246^ Gastrointestinal comorbidities of COVID-19 include hypomotility-related complications, gastrointestinal bleeding, and bowel ischemia.^247^ Gastrointestinal symptoms such as nausea, vomiting, diarrhea, and abdominal pain may precede or accompany with pulmonary symptoms in COVID-19 patients, and the incidence ranged from ~10 to 60%.^247–250^ The three most common symptoms were anorexia, diarrhea, and nausea or vomiting from a meta-analysis comprising 60 studies with 4243 patients.^248^ Anosmia and ageusia were commonly associated with nausea and anorexia after controlling for potential confounders.^251^

Gastrointestinal symptoms were more frequent in critically ill patients with COVID-19 than critically ill patients without COVID-19.^252^ Patients with COVID-19 who had diarrhea required more ventilator support and intensive care than those without diarrhea.^250^ The time from disease onset to admission in COVID-19 patients with gastrointestinal symptoms was longer than in those without gastrointestinal symptoms.^246^ The presence of gastrointestinal symptoms was associated with a high risk of ARDS, non-invasive mechanical ventilation and tracheal intubation, but not with mortality in COVID-19 patients.^253^ Patients with gastrointestinal symptoms had higher rates of positive results for a COVID-19 test than those without.^254^ Roughly 10% of COVID-19 patients presented initially with only gastrointestinal complaints without any respiratory symptoms, which may possibly cause a delay in COVID-19 diagnosis.^246,255^

#### Pathogenesis of gastrointestinal complications of COVID-19

Gastrointestinal injury associated with SARS-CoV-2 infection may be attributed to several proposed mechanisms, including direct cytotoxic damage, intestinal endothelial cell injury and thromboinflammation, dysregulated immune response.^10^ These mechanisms can interact with each other and in turn exacerbate gastrointestinal injury.^10^

The detection of SARS-CoV-2 RNA and viral protein in gastric, duodenal, and rectal glandular epithelial cells^256^ is indictive of the tropism of SARS-CoV-2 to the digestive system. Patients with diarrhea had higher positive rate for SARS-CoV-2 RNA in fecal samples than those without diarrhea.^248^ Current evidence shows that the gastrointestinal symptoms in COVID-19 may be caused by the direct effects of SARS-CoV-2 on the gastrointestinal tract. SARS-CoV-2 may invade the digestive system through ACE2, and growing evidence supports the possible fecal-oral transmission route of SARS-CoV-2. ACE2 is abundantly present in the gastrointestinal epithelial cells, with the highest expression in the small intestine, suggesting that the gastrointestinal tract may be susceptible to SARS-CoV-2 infection.^256–258^ TMPRSS2 had relatively high expression levels in both the small intestine and the colon.^259^ SARS-CoV-2 downregulates ACE2 expression by binding its S protein, thereby contributing to inflammation and injury of gastrointestinal epithelium.^260–262^ ACE2 deficiency in intestinal epithelial cells may be linked to malabsorption of nutrients, altered gut microbiota composition, and intestinal barrier dysfunction.^263^

The activation of coagulation promotes thrombin generation, activates complement system and inhibits fibrinolysis, which triggers thromboinflammation, leading to microthrombi deposition and microvascular dysfunction in the gastrointestinal system.^94^ COVID-19-related cytokine storm and hyperinflammatory immune state might induce gut mucosal immune system activation and enhance immune-mediated inflammatory response in the gastrointestinal system, which contribute to gastrointestinal injury.^94,260,264,265^ The gut microbiota plays a critical role in the maintenance of intestinal homeostasis, and altered microbiota composition is associated with intestinal inflammation. Evidences suggest that SARS-CoV-2 infection is associated with alterations in the gut microbiota.^266^ Gut microbiota may be involved in the magnitude of COVID-19 severity through modulation of host immune responses. Moreover, after resolution of COVID-19, the gut microbiota dysbiosis may be associated with persisting symptoms.^267^ The pathogenesis of the gut microbiota dysbiosis is multifactorial, possibly involving epithelial dysfunction, impaired production of antimicrobial peptide, as well as cytokine storm.^266^

### Hepatobiliary complications

#### Adverse hepatobiliary events of COVID-19

Abnormal liver function tests have been frequently observed in COVID-19 patients, indicating that the liver is one of the most commonly affected extrapulmonary organs by SARS-CoV-2. Clinical case studies show that liver dysfunction is associated with increased risk of mortality in COVID-19 patients. The prevalence of liver injury ranged from 14.8 to 55% in COVID-19 patients.^93,131,132,268–271^ The pooled prevalence of liver function abnormalities was 19%.^272^ In a cohort including 2273 SARS-CoV-2-infected patients, acute liver injury is common but generally mild.^273^ Liver function abnormalities mainly manifest as slightly elevation in levels of alanine aminotransferase (ALT), total bilirubin (TBIL), and gamma-glutamyl transpeptidase (GGT).^274^ Aspartate aminotransferase (AST)-dominant elevation may be earlier, more frequent and significant in patients with severe COVID-19. AST levels showed the strongest correlation with mortality than other indicators of liver injury such as ALT, TBIL, and alkaline phosphatase (ALP) in COVID-19 patients.^275,276^ However, COVID-19 associated severe acute hepatitis has been rarely reported.^277,278^

It is noteworthy that liver dysfunction is closely correlated with disease severity of COVID-19. Patients with severe COVID-19 had higher prevalence of liver injury,^1,131,185^ and patients with liver dysfunction were at higher risks of disease progression.^269,271,279^ The incidence rate of liver injury in deceased patients with COVID-19 was 78%.^274^ Liver failure is observed in COVID-19 deaths and occurs more frequently among critically ill patients.^280^ Of 141 critically ill COVID-19 patients during their ICU stay, 4% developed acute acalculous cholecystitis and 1% developed acute pancreatitis.^281^ Patients with severe liver injury are more likely to have severe clinical course with high risk of mortality.

Patients with preexisting liver diseases such as non-alcoholic fatty liver disease,^282,283^ cirrhosis^284–286^ are more susceptible to SARS-CoV-2 infection and have worse clinical outcome. Chronic hepatitis B and C were more common in patients with liver injury than those without.^287^

#### Pathogenesis of hepatobiliary complications of COVID-19

Underlying mechanisms may be systemic hyperinflammation induced by cytokine storm, pneumonia-associated hypoxia, viral infection in hepatocytes or cholangiocytes and drug-induced liver injury. The cytokine storm may initiate a violent attack to the host and result in liver injury. Dramatical increase in a wide range of proinflammatory cytokines and chemokines such as GM-CSF and IL-6 was observed in patients with liver dysfunction than those with normal liver function.^287^ The liver biopsy showed that COVID-19-associated liver injury was likely immune-mediated.^173^ Taken together, the excessive inflammatory response triggered by SARS-CoV-2 infection may provoke liver injury.

Hypoxemia due to ARDS, systemic inflammatory response syndrome, dysfunction of other organs can contribute to ischemia or reperfusion-induced liver dysfunction in patients with COVID-19. Hypoxia-induced hepatocyte death and production of inflammatory cytokines can be found in hepatic ischemia/reperfusion models.^288^ Moreover, histopathological findings of the liver in COVID-19 patients showed the watery degeneration of a few hepatocytes, which was probably due to ischemia and hypoxia.^269^

SARS-CoV-2 was detected in a proportion of liver biopsy specimens in COVID-19 patients,^289^ but it remains unclear whether SARS-CoV-2 directly infects hepatocytes or cholangiocytes via ACE2. The upregulation of ACE2 expression in the liver was caused by compensatory proliferation of hepatocytes derived from the bile duct epithelial cells in a mouse model of acute liver injury. Some neonatal hepatocytes expressed ACE2 and were susceptible to SARS-CoV-2 infection during this compensatory process.^290^ Pathological and electron microscopic findings revealed typical coronavirus particles in the cytoplasm of hepatocytes from two cases of COVID-19.^291^ Histologically, the predominant histological features of SARS-CoV-2-infected liver were massive apoptosis and binuclear hepatocytes. The GGT and ALP levels were elevated in deceased patients, which may indicate biliary tract injury. All the aforementioned findings suggest that liver injury may not only involve hepatocyte damage but also cholangiocyte dysfunction in patients with COVID-19.

Drug-induced liver injury may also account for some hepatobiliary complications in COVID-19.^292^ Antipyretic therapy is frequently prescribed in COVID-19 patients. Acetaminophen may induce significant liver damage or even cause liver failure in a dose-dependent mechanism.^293^ In clinical practice, multiple drugs including antivirals,^294^ steroids and antibiotics were commonly prescribed in COVID-19 patients, particularly those with severe and critically ill disease.^295^ Some of these drugs may have potential hepatotoxicity and result in liver dysfunction.

### Neurological and psychiatric complications

#### Adverse neurological and psychiatric events of COVID-19

Neurological manifestations of COVID-19 including the central nervous system (CNS)-associated and peripheral nervous system (PNS)-associated ones were present in 18.1–82.0% of the patients. The neurological symptoms were more common in those with severe COVID-19.^12,296–298^ COVID-19 has been reported to be associated with increased risk of mental health disorders,^299^ such as depression, anxiety, schizophrenia, phobia,^300^ obsessive–compulsive symptoms,^301^ post-traumatic stress disorder (PTSD).^302^ A significant proportion of patients experienced psychopathological complications, including 42% of anxiety, 31% of depression, 28% of PTSD, 20% of obsessive–compulsive symptoms, and 40% of insomnia.^303^ The neurological and psychiatric complications of COVID-19 involve encephalitis, cerebral infarction, delirium, Guillain–Barré syndrome,^304–307^ Miller Fisher syndrome,^308^ myopathy, neuromuscular disorders, cephalgia, etc.^309^ Frontline health workers during the COVID-19 pandemic have displayed symptoms of anxiety, depression, insomnia.^310,311^ Long-term isolation triggers mental disorders such as depression and anxiety in some individuals.^312^

#### Pathogenesis of neurological and psychiatric complications of COVID-19

There are many potential gateways of SARS-CoV-2 neuroinvasion from the periphery to the brain. The expression of ACE2 is relatively high in certain brain locations, such as the paraventricular nuclei of the thalamus and choroid plexus.^313,314^ ACE2 is also expressed on the ventrolateral medulla and the nucleus of the tractus solitaries, areas involved in the regulation of the respiratory cycle. This suggests that the virus may affect neurons regulating breathing.^315^ Coronavirus may directly infect sensory neurons in the olfactory epithelium and then spread to CNS from olfactory neurons.^313,316^ NRP1 is expressed of the olfactory epithelium, and can facilitate SARS-CoV-2 cell entry and infectivity.^27^ Moreover, the capillary blood vessels and lymphatics are abundant in the nasal mucosa, which may favor virus invasion.^317,318^ SARS-CoV-2 may possibly invade the brain from the bloodstream through the impaired blood–brain barrier^319^ and leak into the interstitial fluid and the cerebral spinal fluid through the intracerebral lymphatic system. SARS-CoV-2 may also enter the fourth ventricle directly through a damaged blood–cerebrospinal fluid barrier.^320^

The association between systemic inflammatory response and neurological or psychiatric diseases reflects that both innate and adaptive arms of immune system may affect the brain.^321,322^ Systemic inflammation leads to acute brain damage with cognitive impairments and psychiatric symptoms indicative of neurodegeneration.^301,323^ Nearly 80% of septic patients with bacteremia develop sepsis-associated encephalopathy^324^ and delirium.^323^ CNS-resident cells such as astrocytes and microglia represent the first line of defense of the CNS against systemic inflammation and infection. Systemic inflammation allows infiltration of various DAMPs into the nervous system, triggering reactive astrogliosis^325^ and microgliosis.^326^ Dystrophic astrocytes and microglia may be involved in the pathological development of neurodegenerative disorders.

Hypoxia inevitably damages the brain. The greatest central fatigue in acute hypoxia occurs when arterial oxygen saturation (SaO2) is ≤75%, a level that coincides with increasing impairments in neuronal activity.^327^ Hypoxia increases ROS production leading to oxidative damage to neural cells.^328^ Excessive ROS production can directly degenerate or modify cellular macromolecules, including membranes, proteins, lipids, and DNA, and result in activation of inflammatory cascade and protease secretion, finally contributing to brain injury.^328^ Brain hypoxia is also directly linked to activation of inflammatory pathways by stimulating hypoxia-inducible factors and the NF-κB signaling cascade, which promote the release of proinflammatory factors.^329^ Severe hypoxia may cause extensive damage to brain structure, leading to cognitive and neurodegeneration defects. Three main mechanisms appear to be responsible for the occurrence of ischemic strokes in COVID-19,^330,331^ including a hypercoagulable state, vasculitis, and cardiomyopathy. COVID-19 can induce an immune-thrombotic and DIC, which can explain for thrombosis on a consumptive basis.^332^ Other studies have suggested that thrombosis occurs in 20–30% of critically ill COVID-19 patients, even with prophylaxis.^333,334^

Stressors exacerbate both systemic inflammation and inflammatory damage to the brain by activating the hypothalamic–pituitary–adrenal axis.^335^ Levels of CRP demonstrate association with levels of depression.^336^ Neuroinflammation is largely associated with several neuropsychiatric and neurocognitive diseases,^337^ including depression, psychosis and neurodegeneration. Depression is a well-known risk factor of dementia, and psychological burden of COVID-19 may increase the neurodegenerative disease rates in the aftermath of the pandemic.^338^

### Endocrine and metabolic complications

#### Adverse endocrine and metabolic events of COVID-19

Endocrine and metabolic systems can also be involved in COVID-19.^339^ Database from Chinese Centers for Disease Control and Prevention (CDC) showed that of 20,982 patients with COVID-19, 5.3% had diabetes.^340^ Among COVID-19 patients with chronic comorbidities, type 2 diabetes was the second most common morbidity (7.4%).^341^ Diabetes is one of the most relevant comorbidities associated with adverse prognosis of COVID-19.^342–344^ A study on 72,314 COVID-19 patients reported that the mortality rate of patients with diabetes was 7.3%, which was higher than those without diabetes (2.3%).^345^ A whole-population study showed that compared with patients without diabetes, the odds ratios for in-hospital COVID-19-related death were 3.51 in those with type 1 diabetes and 2.03 with type 2 diabetes.^346^ Pregnant women with diabetes might be more vulnerable to the severe effects of COVID-19.^347^

The resultant complications including hyperglycemia and diabetic ketoacidosis were associated with poor prognosis of COVID-19 patients. Acute hyperglycemic crisis, diabetic ketoacidosis and hypertonic hyperglycemia are serious acute metabolic complications usually caused by infection.^348^ Of 2366 patients hospitalized with COVID-19, 157 (6.6%) patients developed diabetic ketoacidosis, 94% of whom had preexisting type 2 diabetes, 0.6% had preexisting type 1 diabetes, and 5.7% patients had no previous diagnosis of diabetes.^349^

Approximately 15% of mild to moderate COVID-19 patients had thyroid dysfunction.^350^ Of 50 COVID-19 patients without previous history of thyroid disease, 56% (28/50) had low thyroid-stimulating hormone (TSH) levels.^351^ The levels of serum TSH and total triiodothyronine (T3) in patients with COVID-19 were significantly lower than in those without COVID-19.^352^ The degree of decrease in TSH and total T3 levels was positively correlated with the disease severity of COVID-19.^351^ Low free T3 due to nonthyroidal illness syndrome is associated with in-hospital mortality in patients in the ICU requiring mechanical ventilation.^353^

#### Pathogenesis of endocrine and metabolic complications of COVID-19

Insulin-producing pancreatic β cells express ACE2 and related entry mediators including TMPRSS2, NRP1, and transferrin receptor (TRFC), with selectively high expression of NRP1.^354^ Evidence demonstrates that SARS-CoV-2 can infect human pancreatic β cell, thereby attenuating the secretion of pancreatic insulin and inducing β cell apoptosis, which possibly contribute to worsening hyperglycemia seen in COVID-19 patients.^354^ Elevated blood glucose levels in COVID-19 patients are related to insulin resistance, which indicates pancreatic β-cell dysfunction or apoptosis, as well as insulin’s inability to dispose of glucose into cellular tissues.^355^ Whether ACE2 was expressed in thyroid tissue or other endocrine organs remains controversial.^356^

Cytokine disorders and T-cell depletion were observed in patients with diabetes, which may be associated with poor clinical outcomes.^97,357–360^ The function of NK cells that play important role in controlling infection is impaired in patients with type 2 diabetes. Glycated hemoglobin was an independent predictor of NK cell activity in patients with type 2 diabetes.^361,362^ In the animal model, hyperglycemia was found to be the main cause of systemic inflammation.^363^ Hyperglycemia and insulin resistance promote synthesis of advanced glycation end products and proinflammatory cytokines, oxidative stress, and adhesion molecules that mediate tissue inflammation,^364^ which may be underlying mechanisms responsible for adverse outcome in patients with diabetes. Potential pathogenetic links between COVID-19 and diabetes include disrupted glucose homeostasis, inflammation, altered immune status and activation of the RAS.^11^ Elevated glucose levels directly induce viral replication and proinflammatory cytokine production, which may favor SARS-CoV-2 infection and monocyte response through hypoxia-inducible factor-1a (HIF-1α)/glycolysis-dependent axis.^365^ Elevated cytokines, imbalance of Th1/Th2 cytokine ratio, decreased peripheral CD8^+^ T cells and NK cell counts contribute to the high mortality of COVID-19 patients with type 2 diabetes.^366^

### Other extrapulmonary complications

#### Co-infection

The prevalence, incidence, and characteristics of existing viral or bacterial co-infection in COVID-19 patients is not well understood and has been raised as a major concern. It was reported in a meta-analysis of 28 studies including 3448 patients with COVID-19 showed that bacterial estimated co-infection was identified in 3.5% of patients and secondary bacterial infection in 15.5% of patients.^367^ The overall proportion of COVID-19 patients with bacterial infection was 7.1% but varied in different patient populations, ranging from 5.8% in hospitalized patients to 8.1% in critically ill cases and up to 11.6% in deceased cases.^367^ The most common organisms reported were Mycoplasma species, *Haemophilus influenzae* and *Pseudomonas aeruginosa*.^367^ Another meta-analysis of 30 studies including 3834 patients reported that 7% of hospitalized COVID-19 patients had a bacterial co-infection.^368^ The pooled proportion of a viral co-infection was 3%, with respiratory syncytial virus and influenza A being the most common pathogens.^368^ Another meta-analysis including 8 studies reported viral co-infections including rhinovirus/enterovirus and influenza A were the most frequent co-infected pathogen. Coronavirus, respiratory syncytial virus, parainfluenza, metapneumovirus, and influenza B virus were also reported as co-pathogens.^369^ To date, the reports on fungal co-infections are scarce or lack of detailed information. A study from China performing fungal culture in all 99 COVID-19 patients at admission confirmed five (5%) cases with fungal infection, including *Aspergillus flavus*, *Candida glabrata*, and *C. albicans*.^370^ Another study reported that 5.8% of the patients had fungal co-infection in 52 critically ill patients, including *A. flavus*, *A. fumigatus*, and *C. albicans*.^371^ A German study showed that COVID-19-associated invasive pulmonary aspergillosis (IPA) was found in five (26.3%) of 19 consecutive critically ill patients with ARDS.^372^ It should be critically paid attention to the probability of COVID-19 accompanied by fungal infections.^373^ The tuberculosis and SARS-CoV-2 co-infection has been rarely reported.^374,375^

A number of immunocompromised individuals were hospitalized with COVID-19 and some were diagnosed with secondary infections.^376^ The specific source and pathogens of these infections have not yet been fully identified. SARS-CoV-2 infection-induced diffuse alveolar injury combined with intra-alveolar neutrophilic infiltration and vascular congestion.^377^ These histologic damages could pave the way for secondary infections including bacterial or fungal infection such as COVID-19-associated invasive pulmonary aspergillosis (CAPA).^373^ Besides, COVID-19 patients are usually characterized by lymphopenia and immune dysfunction, which also help facilitate pathogen invasion.^94^ A case control study reported that steroids use was also a significant risk factor for bacterial infection in patients with severe to critically ill COVID-19.^378^ Critically ill patients were more likely to develop fungal co-infections.^371^

#### Ocular complication

According to a systematic review including 4432 patients from 35 studies, the prevalence rate of ocular manifestations was 11.3% in adult patients with COVID‐19.^379^ Ocular manifestations are non-specific, and conjunctivitis manifested as redness, watering, discharge, and foreign body sensations, is the most commonly reported.^380^ Other ocular complications include dry eye, blurred vision, ocular pain, photophobia and itchiness, etc. Notably, ocular signs and symptoms were the initial presentation in 3.3% COVID-19 patients.^379^ Patients with severe pneumonia have a significantly higher likelihood of ocular manifestations than mild-to-moderate pneumonia.

The conjunctiva is directly exposed to the environment, and easily contaminated with respiratory droplets or hands carrying the virus. A pooled data showed that the positive rate of SARS-CoV-2 RNA was 7.4% in the ocular surface of COVID-19 patients.^379^ Several studies have already demonstrated the expressions of ACE2 and TMPRSS2 in the cornea and conjunctiva, although their expressions were obviously lower in comparison to other tissues, such as lung and digestive tract.^381^ In vitro study demonstrated that SARS-CoV-2 can directly infect the corneal cells from human eyes and hESC-derived eye organoids.^382^ Although conjunctiva is unlikely to be a preferred entry gateway for SARS-CoV-2, the expressions of SARS-CoV-2 in tears and conjunctival secretions partially explain ocular complications.

#### Ear-nose-throat (ENT) complication

Mounting evidence indicates that olfactory and gustatory dysfunction is closely correlated with COVID-19.^383^ A systematic review summarized that olfactory and gustatory loss was observed in 41.0% and 38.2% of COVID-19 patients, respectively.^384^ In particular, some patients may only have olfactory or gustatory loss in the absence of other clinical symptoms. A multicenter study from Europe reported that 11.8% of patients presented with olfactory loss as their first symptoms.^385^ Most patients get recovery from the symptoms within 4–6 weeks of follow-up, and only 3.59% and 3.27% of patients with olfactory and gustatory loss, respectively, showed partial recovery beyond 8 weeks.^386^

The viruses may induce an inflammatory response of nasal mucosa or directly damage the olfactory neuroepithelium. SARS-CoV-2 may not directly enter olfactory sensory neurons due to lacking of ACE2 receptor expression, but rather attack the supporting and stem cells of olfactory epithelium expressing ACE2 receptor.^387^ COVID-19 patients with influenza-like illness displayed the increased frequency of olfactory loss.^386^ However, olfactory loss was also reported in COVID-19 patients without nasal symptoms or significant inflammation.^388^

#### Dermatologic complication

Cutaneous manifestations have been reported in 1.8% to 20.4% of COVID-19 patients.^389^ The appearance of skin varies, including maculopapular rashes, urticaria, petechiae/purpura, vesicles, chilblains, livedo racemosa, and distal ischemia or necrosis.^390^ The trunk is a prone area of skin lesions, but the involvement of extremities may also occur. Most of the skin lesions are self-resolving, and do not appear to be related to the disease severity. Cutaneous involvement may be primarily attributed to an immune response to viral protein or nucleotides, or vasculitis and thrombotic vasculopathy secondary to systemic consequences caused by COVID-19.^391^ Pathological examination revealed the existence of pauci-inflammatory thrombogenic vasculopathy in the purpuric skin lesions, and the colocalization of SARS-CoV-2 S protein with C4d and C5b-9 in both normally-appearing and grossly involved skin.^392^

#### Reproductive complication

A concern has been raised that SARS-CoV-2 may cause damage to testis, and even result in the infertility. In patients with COVID-19, luteinizing hormone (LH) and follicle-stimulating hormone (FSH) levels were elevated, while testosterone and dihydrotestosterone levels markedly decreased.^393^ In recovered male patients, the count, concentration and motility of sperm were still declined slightly, although sexual hormones have returned to normal levels.^394^ Patients with a longer recovery time showed poorer sperm quality. Pathological studies conducted on deceased COVID-19 patients found vacuolation and detachment from the tubular basement membrane of Sertoli cells, the destruction of seminiferous tubules, the reduction of Leydig cells, and the inflammatory infiltrate of T lymphocytes in the interstitium.^395^ ACE2 and TMPRSS2 expression is abundant in spermatogonial cells, interstitial cells, and supporting cells of testis, suggesting the testis as a potential target of SARS-CoV-2 infection.^396^ The immune responses triggered by SARS-CoV-2 produce lots of inflammatory mediators and induce oxidative stress in testicular cells, potentially damaging the DNA of spermatozoa. In addition, SARS-CoV-2 causes damage to Leydig cells, and subsequently lowers testosterone secretion, which may ultimately disrupt the process of spermatogenesis.^397^

There is no evidence that pregnancy and childbirth alter susceptibility to SARS-CoV-2 infection. These studies did not report severe maternal complications in pregnant women with COVID-19. A few studies have revealed an increased risk of preterm birth and cesarean delivery, but it is unclear whether these results are directly related to SARS-CoV-2.^398^ There is no direct data supporting mother-to-child transmission of SARS-CoV-2, but newborns of COVID-19 infected mothers have tested positive for SARS-CoV-2-specific antibodies and were also presenting with increased IL-6 levels.^399^

#### Hematopoietic system complication

The hematopoietic system produces immune cells that can defeat viral infections and is a source of hematopoietic stem cells (HSC) and progenitor cells (HPC). Human HSC and HPC express ACE2 on the cell surface, making them susceptible to SARS-CoV-2 infection. SARS-CoV-2 S protein binds to ACE2, induces defects in human HPC colony-forming ability and inhibits the expansion of HSC and HPC subpopulations in vitro.^400^ In addition, in human very small embryonic stem cells (VSELs) and HSCs, the interaction of ACE2 with S protein activates the NLRP3 inflammasome, which may cause cell pyrolysis.^401^ The plasma of severe COVID-19 patients induces HPC to produce suppressive bone marrow cells in vitro, in relation to the high levels of IL-6 and IL-10 in plasma.^402^

The hemoglobin level of patients with severe COVID-19 significantly decreased, but the circulating nucleated red blood cells increased. SARS-CoV-2 may directly infect human erythroid progenitor cells, resulting in the formation and expansion of erythroid progenitor cell colonies, thereby increasing stress erythropoiesis.^403^

Multiomics analysis revealed increased proliferation and metabolic hyperactivity of plasmablasts in peripheral blood of patients with severe COVID-19, as well as IFN-activated circulating megakaryocyte expansion and increased erythrocyte production characterized by hypoxic signaling.^404^ Another study has shown that there is a tendency for myeloid skewing in circulating HSCs and HPCs in patients with COVID-19, and the frequency of common lymphoid progenitor cells is lower in severe patients, while granulocyte/macrophage progenitor cells/neutrophil-like cells appear in severe and fatal cases.^405^

## The implication for therapeutics

Currently, antivirals, glucocorticoids, and immunoglobulin treatments are still debating for their effectiveness of significant improvement in the survival of patients with severe COVID-19. The unconstrained host inflammatory response is the main driver of the pathology of severe COVID-19. For COVID-19 in the acute setting, 6 mg daily of dexamethasone (equivalent to 40 mg of prednisone) for 10 days reduced mortality from 25·7% to 22·9%, The results were more striking in patients requiring oxygen or invasive ventilation.^406^ However, chronic glucocorticoids increased the odds of hospitalization for COVID-19 in patients with rheumatic disease.^407^ Systemic glucocorticoids increased the odds of COVID-19 related death in patients with inflammatory bowel disease.^408^ It is proposed that dexmedetomidine should be considered in COVID-19 patients admitted to ICU when sedation is required, during the early disease course to help prevent the onset or progression of multiorgan dysfunction.^409^ Further clinical studies are warranted to optimize the individual strategies with these medications.

**Fig. 4:**
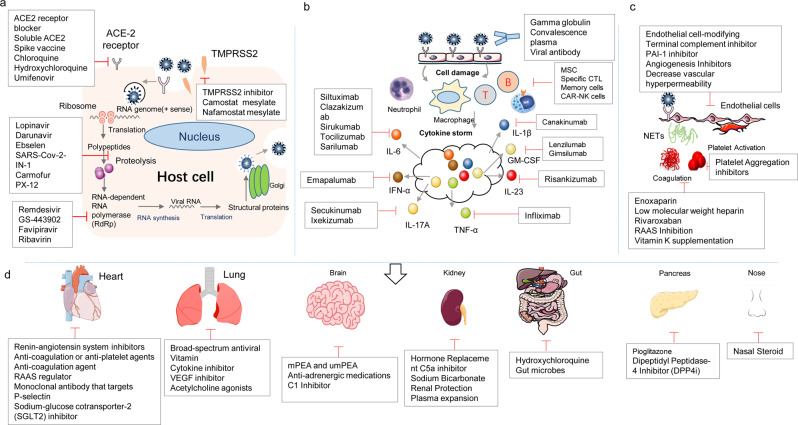
Potential therapeutic targets against COVID-19 which may be involved in virus replication, immune response, vascular endothelial coagulation system and important organs related complications. **a** SARS-CoV-2 virus invades host cells. Viral replication involves multiple steps: attachment, penetration, uncoating, replication, assembly, and release. **b** Viral infection induces an antiviral response, recruiting innate and adaptive immune cells such as macrophages, neutrophil, dendritic cells, T cells, B cells, and NK cells, and leads to cytokine storm. **c** SARS-CoV-2 invades endothelial cells and affects the coagulation system, increasing the risk of embolism and bleeding. **d** These host–virus interaction may affect multiple important organs and cause severe complications. The therapeutic targets of these steps are shown in this figure

Alternatively, targeting the key mechanisms responsible for the pathogenesis of COVID-19, including viral entry and replication, cytokine storm, lymphopenia and endothelial damage and thromboinflammation, may be promising treatment strategies for severe COVID-19 (Fig. 4 and Table 1). Potential anti- SARS-CoV-2 treatments can be divided into two categories depending on the target, one is acting on the host cells or immune system, and the other is on SARS-CoV-2 itself.

**Table 1:** Clinical studies of potential therapeutic options for COVID-19

Target: organs or systems	Pathological phenomenon	Mechanism of action	Treatment	Clinical trial	Country	Number of patients	Trial phase
Virus	Virus entry into the host and binding with the host cell receptor	Blocking receptor-binding domain	Ensovibep	NCT04870164	United Kingdom	24	Phase 1
Virus	Virus entry into the host and binding with the host cell receptor	TMPRSS2 inhibitor	Camostat Mesilate	NCT04470544	United States	264	Phase 2
Virus	Virus entry into the host and binding with the host cell receptor	TMPRSS2 inhibitor	Camostat Mesilate	NCT04583592	United States	295	Phase 2
Virus	Virus entry into the host and binding with the host cell receptor	Block viral entry	RhACE2 APN01	NCT04335136	Turkey	185	Phase 2
Virus	Viral replication and clearance	Antiviral activity	Niclosamide	NCT04558021	Turkey	200	Phase 3
Virus	Viral replication and clearance	Broad antiviral activity	Peg-IFN Lambda	NCT04534673	United States	40	Phase 2
Virus	Viral replication and clearance	Broad antiviral activity	Remdesivir	NCT04560231	Pakistan	30	Phase 1
Virus	Viral replication and clearance	Broad antiviral activity	Remdesivir	NCT04738045	Egypt	90	Phase 4
Virus	Viral replication and clearance	Broad antiviral activity	Favipiravir	NCT04499677	United Kingdom	240	Phase 2
Virus	Viral replication and clearance	Broad antiviral activity	Lopinavir/Ritonavir	NCT04466241	Côte D’Ivoire	294	Phase 3
Virus	Viral replication and clearance	Inhibit viral replication	Amantadine	NCT04952519	Poland	500	Phase 3
Virus	Viral replication and clearance	Inhibit viral replication	Amantadine hydrochloride	NCT04854759	Poland	200	Phase 3
Virus	Viral replication and clearance	Interference with viral proliferation	leflunomide	NCT04361214	United States	20	Phase 1
Virus	Viral replication and clearance	Antiviral activity	Nafamostat Mesilate	NCT04390594	Senegal	186	Phase 3
Virus	Viral replication and clearance	Antiviral activity	Niclosamide	NCT04753619	Iraq	150	Phase 2
Virus	Viral replication and clearance	Broad antiviral activity	Favipiravir	NCT04425460	China	256	Phase 3
Virus	Viral replication and clearance	Broad antiviral activity	Sofosbuvir	NCT04535869	Egypt	50	Phase 3
Virus	Viral replication and clearance	Broad antiviral activity	Ribavirin	NCT04828564	Turkey	100	Phase 2
Virus	Viral replication and clearance	Inhibit viral replication	Chlorpromazine (CPZ)	NCT04366739	France	40	Phase 3
Virus	Viral replication and clearance	Inhibit viral replication	Chlorpromazine	NCT04354805	Egypt	100	Phase 3
Virus	Viral replication and clearance	Antiviral activity	Nafamostat Mesylate	NCT04418128	South Korea	84	Phase 3
Virus	Viral replication and clearance	Broad antiviral activity	Favipiravir	NCT04400682	Turkey	30	Phase 1
Virus	Viral replication and clearance	Broad antiviral activity	Remdesivir	NCT04280705	United States	1062	Phase 3
Virus	Viral replication and clearance	Broad antiviral activity	Remdesivir	NCT04401579	United States	1033	Phase 3
Virus	Viral replication and clearance	Broad antiviral activity	Remdesivir	NCT04345419	Egypt	200	Phase 2
Virus	Viral replication and clearance	Broad antiviral activity	Favipiravir	NCT04407000	Turkey	30	Phase 1
Virus	Viral replication and clearance	Broad antiviral activity	Lopinavir and ritonavir	NCT04252885	China	86	Phase 4
Virus	Viral replication and clearance	Broad antiviral activity	Lopinavir/ritonavir	NCT04276688	Hong Kong	127	Phase 2
Virus	Viral replication and clearance	Interference with viral proliferation	LAU-7b: fenretinide	NCT04417257	United States	240	Phase 2
Virus	Viral replication and clearance	Broad antiviral activity	Favipiravir	NCT04501783	Russian Federation	168	Phase 3
Virus	Viral replication and clearance	Interference with viral proliferation	Leflunomide	NCT05007678	United Kingdom	178	Phase 3
Virus	Viral replication and clearance	Antiviral activity	Niclosamide	NCT04399356	United States	73	Phase 2
Virus	Viral replication and clearance	Broad antiviral activity	Favipiravir	NCT04359615	Iran	40	Phase 4
Virus	Viral replication and clearance	Broad antiviral activity	Sofosbuvir	NCT04530422	Egypt	250	Phase 3
Immune system	Cytokine storm	IL-6 Antagonist	Siltuximab, tocilizumab	NCT04486521	Saudi Arabia	860	−
Immune system	Cytokine storm	IL-6 inhibitor	Clazakizumab	NCT04659772	USA	1	Phase 2
Immune system	Cytokine storm	IL-6 inhibitor	Clazakizumab	NCT04343989	USA	180	Phase 2
Immune system	Cytokine storm	Anti-IL-6 immunoglobulin G1 kappa (IgG1k) monoclonal antibody (mAb)	Sirukumab	NCT04380961	USA	212	Phase 2
Immune system	Cytokine storm	Anti-IL-6 receptor antibody	Tocilizumab	NCT04730323	Pakistan	93	Phase 4
Immune system	Cytokine storm	Anti-IL-6 receptor antibody	Sarilumab	NCT04661527	Spain	60	Phase 2
Immune system	Cytokine storm	Interferon gamma blocking antibody	Emapalumab	NCT04324021	USA	16	Phase 2 Phase 3
Immune system	Cytokine storm	Interleukin-IL-17A Antagonist	Secukinumab	NCT04403243	Russia	70	Phase 2
Immune system	Cytokine storm	IL-17 inhibitor	ixekizumab	NCT04724629	Brazil	60	Phase 3
Immune system	Cytokine storm	Tumor necrosis factor inhibitors	Infliximab	NCT04734678	Egypt	84	−
Immune system	Cytokine storm	IL-1β inhibitor	Canakinumab	NCT04362813	USA	454	Phase 3
Immune system	Cytokine storm	GM-CSF inhibitor	Lenzilumab	NCT04351152	USA	520	Phase 3
Immune system	Cytokine storm	Monoclonal antibody against GM-CSF	Gimsilumab	NCT04351243	USA	227	Phase 2
Immune system	Cytokine storm	IL-23 inhibitor	Risankizumab	NCT04583956	USA	1	Phase 2
Immune system	Immunomodulatory	Immune support	Biological: HB-adMSCs	NCT04348435	USA	55	Phase 2
Immune system	Immunomodulatory	Specific cytotoxic T lymphocytes	SARS-CoV-2 antigen-specific cytotoxic T lymphocyte	NCT04742595	USA	16	Phase 1
Immune system	Immunomodulatory	Immunomodulatory	RAPA-501-Allo off-the-shelf therapy of COVID-19	NCT04482699	USA	88	Phase 1 Phase 2
Immune system	Immunomodulatory	Immunomodulatory	NKG2D-ACE2 CAR-NK cells	NCT04324996	China	90	Phase 1 Phase 2
Immune system	Immunomodulatory	Reduce the proinflammatory state and promoting the regeneration of damaged tissues	MSC	NCT04611256	Mexico	20	Phase 1
Immune system	Immunomodulatory	Immunomodulatory	T memory cells and NK cells	NCT04578210	Spain	58	Phase 1 Phase 2
Immune system	Immunomodulatory	Regulates inflammation and immunity	Infusion IV of Mesenchymal Stem cells	NCT04416139	Mexico	10	Phase 2
Immune system	Anti-inflammatory effects	Anti-inflammatory effects	Autologous activated platelet-rich plasma	NCT04715360	Indonesia	30	Phase 1 Phase 2
Immune system	Anti-inflammatory effects	Inhibit the inflammatory response	UC-MSCs	NCT04339660	China	30	Phase 1 Phase 2
Immune system	Anti-inflammatory effects	Reduce lung inflammation and pathological impairment	MSC exosome inhalation	NCT04491240	Russia	30	Phase 1 Phase 2
Immune system	Immunomodulatory	Immune-mediated inflammatory	Mesenchymal stromal cells	NCT04361942	Spain	24	Phase 2
Immune system	Immunomodulatory	Virus neutralization. Other possible mechanisms include antibody-dependent cytotoxicity and phagocytosis	Convalescent plasma	NCT04476888	Pakistan	110	−
Immune system	Immunomodulatory	Prevent or shut down the continuous inflammatory response caused by the virus	COVID-19 convalescent plasma	NCT04374526	Italy	29	Phase 2 Phase 3
Immune system	Immunomodulatory	Improve high inflammation state and respiratory function	Therapeutic plasma exchange	NCT04751643	France	132	−
Immune system	Immunomodulatory	Immunomodulatory	Intravenous immunoglobulin (IVIG)	NCT04500067	Ukraine	76	Phase 3
Immune system	Immune reconstitution	Immune reconstitution	Recombinant interleukin-7 (CYT107)	NCT04442178	USA	48	Phase 2
Immune system	Immunomodulatory	Prevent or shut down the continuous inflammatory response caused by the virus	COVID-19 convalescent plasma (CCP)	NCT04421404	USA	42	Phase 2
Immune system	Immunomodulatory	Immunomodulatory	Monoclonal antibody to S protein of SARS-CoV-2	NCT04840459	USA	1000	Phase 2
Immune system	Immunomodulatory	Immunomodulatory	SARS-CoV-2 antibody-based IVIG therapy	NCT04521309	Pakistan	50	Phase 1 Phase 2
Immune system	Immunomodulatory	Immunomodulatory	JS016 (anti-SARS-CoV-2 monoclonal antibody)	NCT04931238	China	200	Phase 1
Immune system	Immunomodulatory	Immunomodulatory	plasma therapy using convalescent plasma with antibody against SARS-CoV-2	NCT04356534	Ireland	40	−
immune system	Cytokine storm	Regulation of the inflammatory cytokine response	Vitamin D, Omega DHA/EPA, vitamin C, vitamin B complex, and zinc acetate	NCT04828538	Mexico	3600	−
Immune system	Immune activation	Suppressor of cytokine activation	Zofin: derived from human amniotic fluid	NCT04384445	USA	20	Phase 1、Phase 2
Immune system	Intense inflammatory cascade	Adjunct immune modulation therapies	Vitamin C	NCT04401150	Canada	800	Phase 3
Immune system	Cytokine storm	Human normal immunoglobulin	IVIG	NCT04500067	Ukraine	76	Phase 3
Immune system	Cytokine storm	Human normal immunoglobulin	Human immunoglobulin	NCT04350580	France	146	Phase 3
Immune system	Cytokine storm	Immunosuppressant	Ciclesonide	NCT04377711	USA	400	Phase 3
Immune system	Cytokine storm	Vitamin	Cholecalciferol	NCT04552951	Spain	80	Phase 4
Immune system	Cytokine storm	Vitamin	Vitamin D3, vitamin C/Zinc, vitamin K2/D	NCT04780061	Canada	200	Phase 3
Immune system	Cytokine storm	Vitamin	Oral 25-hydroxyvitamin D3	NCT04386850	Islamic Republic	1500	Phase 2,3
Immune system	Cytokine storm	Mesenchymal stem cells	HB-adMSCs	NCT04348435	USA	55	Phase 2
Immune system	Cytokine storm	Bone marrow-derived extracellular vesicles	DB-001	NCT04493242	USA	120	Phase 2
immune system	Elevated numbers of neutrophils	NETs degradation	rhDNase I	NCT04409925	Canada	25	Phase 1
Endothelial	Pulmonary edema	Abl2/Arg inhibitors	Imatinib	NCT04794088	Netherlands	90	Phase 2
Endothelial	Acute respiratory distress syndrome	DNase inhibitors	Dornase alfa	NCT04355364	France	100	Phase 3
Endothelial	Excessive blood clotting	Vasodilator and inhibitor of platelet aggregation	Dipyridamole	NCT04391179	United States	100	Phase 2
Endothelial	Endothelial dysfunction	Endothelial cell modifying	Defibrotide	NCT04652115	United States	42	Phase 2
Endothelial	Endothelial dysfunction	Endothelial cell modifying	Defibrotide	NCT04348383	Spain	150	Phase 2
Endothelial	Complement-mediated diseases	Terminal complement inhibitor	Eculizumab	NCT04346797	France	120	Phase 2
Endothelial	Endothelial dysfunction	Increase NO production and release	Atorvastatin + l-arginine + folic acid + nicorandil + nebivolol	NCT04631536	Lebanon	80	Phase 3
Endothelial	Endothelial injury	PAI-1 inhibitor	TM5614	NCT04634799	United States	80	Phase 2
Endothelial	Microvascular endothelial dysfunction	Platelet aggregation inhibitors	Iloprost	NCT04420741	Denmark	80	Phase 2
Endothelial	Vascular endothelial dysfunction	Restore endothelial glycocalyx and Inhibit thrombosis	Suloexide	NCT04483830	Mexico	243	Phase 2 Phase 3
Endothelial	Endothelial injury	C5a inhibitor	Ravulizumab	NCT04570397	United States	32	Phase 3
Endothelial	Vascular dilation	Angiogenesis inhibitors	BEVACIZUMAB	NCT04822818	France	174	Phase 3
Endothelial	Vascular leakage	decrease vascular hyperpermeability	FX06	NCT04618042	France	50	Phase 2
Coagulation system	Higher hypercoagulability	Anticoagulation	Enoxaparin	NCT04360824	USA	170	Phase 4
Coagulation system	Higher hypercoagulability	Anticoagulation	Enoxaparin, unfractionated heparin, atorvastatin, matched placebo	NCT04486508	Iran	600	Phase 3
Coagulation system	Higher hypercoagulability	Anticoagulation	Enoxaparin	NCT04354155	USA	40	Phase 2
Coagulation system	Higher hypercoagulability	Anticoagulation	Low-molecular-weight heparin, fondaparinux	NCT04359212	Italy	90	–
Coagulation system	Higher hypercoagulability	Anticoagulation	Enoxaparin	NCT04408235	Italy	300	Phase 3
Coagulation system	Higher hypercoagulability	Anticoagulation	Tinzaparin, unfractionated heparin	NCT04344756	France	808	Phase 2
Coagulation system	Higher hypercoagulability	Anticoagulation	Enoxaparin	NCT04345848	Switzerland	200	Phase 3
Coagulation system	Higher hypercoagulability	Anticoagulation	Enoxaparin, heparin	NCT04359277	USA	77	Phase 3
Coagulation system	Higher hypercoagulability	Anticoagulation	Low-molecular-weight heparin (LMWH), unfractionated heparin (UFH)	NCT04362085	Canada	465	Phase 3
Coagulation system	Higher hypercoagulability	Anticoagulation	Enoxaparin	NCT04366960	Italy	189	Phase 3
Coagulation system	Higher hypercoagulability	Anticoagulation	Enoxaparin, heparin, lovenox	NCT04367831	USA	100	Phase 4
Coagulation system	Higher hypercoagulability	Anticoagulation	Heparin	NCT04372589	USA	1200	Phase 3
Coagulation system	Higher hypercoagulability	Anticoagulation	Enoxaparin	NCT04373707	France	602	Phase 4
Coagulation system	Higher hypercoagulability	Anticoagulation	Low-molecular-weight heparin	NCT04393805	Italy	744	–
Coagulation system	Higher hypercoagulability	Anticoagulation	Rivaroxaban, enoxaparin	NCT04394377	Brazil	615	Phase 4
Coagulation system	Higher hypercoagulability	Anticoagulation	Enoxaparin	NCT04401293	USA	257	Phase 3
Coagulation system	Higher hypercoagulability	Anticoagulation	Rivaroxaban	NCT04416048	Germany	400	Phase 2
Coagulation system	Higher hypercoagulability	Platelet inhibition	Tirofiban, clopidogrel, acetylsalicylic acid, fondaparinux	NCT04368377	Italy	5	Phase 2
Coagulation system	Reduced vitamin K status	Vitamin K supplementation	Vitamin K2 in the form of menaquinone-7 (MK-7), placebo	NCT04770740	Netherlands	40	Phase 2
Cardiovascular	Dysfunction of RAAS	Recover the function of ACE2-RAAS	Recombinant bacterial ACE2 receptors-like enzyme of B38-CAP (rbACE2)	NCT04375046	China	24	Phase 1
Cardiovascular	Dysfunction of RAAS	RAAS inhibition	RAAS inhibitor	NCT04508985	Canada	40	/
Cardiovascular	Cardiovascular disease or risk factors	Anti-inflammatory	Cannabidiol	NCT04615949	Arizona, USA	422	Phase 2、Phase 3
Vascular endothelial system	Coagulation disorders	Anticoagulant	Enoxaparin Atorvastatin	NCT04486508	Islamic Republic	600	Phase 3
Circulatory system	Vascular endothelial injury, cytokine storm	FXa inhibitor, HMG-CoA inhibitor	Apixaban, Atorvastatin	NCT04801940	UK	2631	Phase 3
Vascular endothelial system	Cytokine storm, vascular endothelial injury	Vitamin C	Vitamin C	NCT04401150	Canada	800	Phase 3
Vascular endothelial system	Vascular endothelial injury	Phyto preparation	Hesperidin	NCT04715932	Canada	216	Phase 2
Cardiac, kidney	multiorgan failure	Sodium–glucose cotransporter-2 (SGLT-2) inhibitor	Dapagliflozin	NCT04350593	USA、Argentina、Brazil、Canada、India、Mexico、UK	1250	Phase 3
Cardiovascular	Acute cardiac injury	Cardioprotective medicines	Aspirin, clopidogrel, rivaroxaban, atorvastatin, omeprazole	NCT04333407	UK	3170	−
Cardiovascular	Thrombotic events	Anticoagulation or anti-platelet agents	Apixaban, aspirin	NCT04498273	USA	7000	Phase 3
Cardiovascular	Thrombotic events	Anti-inflammatory and antithrombotic function	Enoxaparin, unfractionated heparin, atorvastatin	NCT04486508	Iran	600	Phase 3
Cardiovascular	Dysregulated immune response	Immunomodulatory and cardiovascular drugs	EDP1815, dapagliflozin and Ambrisentan	NCT04393246	UK	1407	Phase 2、Phase 3
Cardiovascular	Cardiac injury	Reduce cardiac injury	Colchicine tablets	NCT04355143	USA	150	Phase 2
Cardiovascular	Thrombotic events	Anticoagulation agent	Rivaroxaban	NCT04757857	Brazil	1000	Phase 4
Cardiovascular	Thrombotic events	Antagonize plasminogen activator inhibitor	TM5614	NCT04634799	USA	80	Phase 1、Phase 2
Cardiovascular	Thrombotic events	Anticoagulation agent	Enoxaparin	NCT04492254	Australia	1370	Phase 3
Cardiovascular	Thrombotic events	Anticoagulation agent	Apixaban	NCT04746339	Brazil	1000	Phase 4
Cardiovascular	Longer-term complications occurring in the convalescent phase	Improve the quality of life	Apixaban, atorvastatin	NCT04801940	UK	2631	Phase 3
Cardiovascular	Thrombotic events	Anticoagulation agent	Rivaroxaban	NCT04416048	Germany	400	Phase 2
Cardiovascular	Endothelial dysfunction	Improve endothelial function	Atorvastatin + l-arginine + folic acid + nicorandil + nebivolol	NCT04631536	Lebanon	80	Phase 3
Cardiovascular	Thrombotic events	Anticoagulation agent	Enoxaparin, apixaban	NCT04512079	USA、Brazil、Colombia、India、Mexico	3600	Phase 4
Cardiovascular	Thrombotic events	Anticoagulation agent	Enoxaparin	NCT04400799	Germany、Switzerland	1000	Phase 3
Cardiovascular	Thrombotic events	Anticoagulation agent	Enoxaparin	NCT04646655	Italy	300	Phase 3
Cardiovascular	Viral injury of the vascular endothelium	Monoclonal antibody that targets P-selectin	Crizanlizumab	NCT04435184	USA	50	Phase 2
Cardiovascular	Myocardial infarction in combination with COVID-19	Antiarrhythmic effect	Atorvastatin, atorvastatin-ezetimibe	NCT04900155	Russian Federation	200	−
Cardiovascular	Thrombotic events	Antithrombotic therapy	Apixaban	NCT04650087	USA	5320	Phase 3
Cardiovascular	Thrombotic events	Thromboprophylaxis	Rivaroxaban	NCT04662684	Brazil	320	Phase 3
Cardiovascular	Thrombotic events	Anticoagulation agent	Enoxaparin	NCT04345848	Switzerland	200	Phase 3
Cardiovascular	Thrombotic events	Anticoagulation agent	Enoxaparin, heparin	NCT04367831	USA	100	Phase 4
Cardiovascular	Thrombotic events	Anticoagulation agent	Enoxaparin	NCT04354155	USA	40	Phase 2
Cardiovascular	Thrombotic events	Anticoagulation therapy	Therapeutic anticoagulation	NCT04362085	Canada	465	Phase 3
Cardiovascular	Thrombotic events	Anticoagulation agent	Thromboprophylaxis	NCT04360824	USA	170	Phase 4
Cardiovascular	Thrombotic events	Anticoagulation agent	Enoxaparin	NCT04373707	France	602	Phase 4
Cardiovascular	Acute pulmonary hypertension (aPH) and/or acute Cor pulmonale (ACP)	Decrease pulmonary arterial pressure	PDNO	NCT04885491/2020-002982-33	Sweden	16	Phase 1、Phase 2
Cardiovascular	Hyperviscosity	Plasma exchange	Therapeutic plasma exchange	NCT04441996	USA	20	Phase 4
Cardiovascular	Thrombotic events	Anticoagulation therapy	Edoxaban, colchicine	NCT04516941	Belgium、Italy、Spain、Switzerland	420	Phase 3
Cardiovascular	Thrombotic events	Anticoagulation therapy	Tinzaparin	NCT04730856	Spain	600	Phase 3
Cardiovascular	Thrombotic events	Anticoagulation therapy	Enoxaparin	NCT04508439	Mexico	130	−
Cardiovascular	Coagulopathy	Anticoagulation therapy	Therapeutic anticoagulation	NCT04444700	Brazil	465	Phase 3
Cardiovascular	Thrombotic events	Anticoagulation agent	Enoxaparin	2020-003125-39	Germany	1370	−
Cardiovascular	Thrombotic events	Anticoagulation agent	Low-molecular-weight heparin	2020-001709-21	France	550	−
Cardiovascular	Thrombotic events	Anticoagulation agent	Enoxaparin	2020-005624-10	Germany	1000	−
Cardiovascular	Downregulation of ACE2	Anti-inflammatory and antithrombotic function-RAAS	Omega3-FA	NCT04658433	Jordan	100	−
Cardiovascular	Cardiovascular disease or risk factors	Renin–angiotensin system inhibitors-RAAS	ACEi、ARB	NCT04591210	Canada, Brazil, Mexico	1155	Phase 3
Cardiovascular	Viral cell invasion	Increase ACE2 expression and improve mechanisms of host defense or hyperinflammation-RAAS	Discontinuation/continuation of ACEi、ARB	NCT04338009	USA	152	−
Cardiovascular	ARDS	RAAS regulator	Telmisartan	NCT04355936	Argentina	400	Phase 4
Cardiovascular	Overaction of RAS	Similar peptide to Ang(1–7)-RAAS	TRV027	NCT04419610	UK	30	Phase 1
Cardiovascular	Viral entry and viral replication	Recombinant human angiotensin-converting enzyme 2 -RAAS	RhACE2 APN01	NCT04335136	Austria、Denmark、Germany、Russian Federation、UK	185	Phase 2
Respiratory system	Host viral entry	TMPRSS2 inhibitors	Nafamostat Mesilate	NCT04352400	Italy	256	Phase 2、Phase 3
Immune and respiratory system	Cytokine storm	Prophylactic corticosteroid	Methylprednisolone	NCT04355247	Puerto Rico	20	Phase 2
Respiratory system	Cytokine storm	Mesenchymal stem cells	Umbilical cord-derived mesenchymal stromal cells	NCT04333368	France	40	Phase 1
Respiratory system	Cytokine storm	Biomarker-tailored steroid	Methylprednisolone	NCT03852537	USA	44	Phase 2
Respiratory system	Viral growth	Broad-spectrum antiparasitic	Ivermectin	NCT04739410	Pakistan	50	Phase 4
Respiratory system	Cytokine storm	Antioxidant	Sodium pyruvate	NCT04871815	USA	50	Phase 2,3
Respiratory system	Cytokine storm	Antihistamines	Cetirizine and famotidine	NCT04836806	USA	160	Phase 4
Respiratory system	Viral growth	Broad-spectrum antiviral	Remdesivir	NCT04978259	Finland	202	Phase 4
Respiratory system	Viral growth	Broad-spectrum antiviral	Favipiravir	NCT04694612	Nepal	676	Phase 3
Respiratory system	Viral growth	Broad-spectrum antiviral	Triazavirin	NCT04973462	Egypt	80	Phase 4
Respiratory system	Viral growth	Broad-spectrum antiviral	Ivermectin	NCT04673214	Mexico	114	Phase 3
Respiratory system	Viral growth	Broad-spectrum antiviral	Lopinavir/ritonavir	NCT04466241	Cote d’Ivoire	294	Phase 2,3
Respiratory system	Host viral entry	Vaccine based on peptide antigens	EpiVacCorona	NCT04780035	Russian Federation	3000	Phase 3
Respiratory system	Viral growth	Broad-spectrum antiparasitic	Nitazoxanide 500 mg oral tablet	NCT04406246	Mexico	150	Phase 4
Respiratory system	Viral growth, cytokine storm	Broad-spectrum antiviral	Ivermectin and doxycycline	NCT04523831	Bangladesh	400	Phase 3
Respiratory system	Viral growth	Broad-spectrum antiviral	Ivermectin tablets, doxycycline tablets	NCT04729140	USA	150	Phase 4
Respiratory system	Cytokine storm	Antioxidant	Sodium pyruvate	NCT04824365	USA	60	Phase 2,3
Respiratory system	Viral growth	RNA polymerase inhibitor	Favipiravir	NCT04600999	Hungary	150	Phase 3
Respiratory system	Viral growth, Cytokine storm	ACE2 inhibitor	Hydroxychloroquine	NCT04354428	USA	300	Phase 2,3
Respiratory system	Host viral entry	Acetylcholine agonists	Nicotine patch	NCT04583410	France	1633	Phase 3
Respiratory system	Viral growth	Broad-spectrum antiparasitic	AVIGAN	NCT04529499	Kuwait	780	Phase 3
Respiratory system	Viral growth	Broad-spectrum antiparasitic	Ivermectin	NCT04646109	Turkey	66	Phase 3
Respiratory system	Viral growth	Broad-spectrum antiparasitic	Favipiravir	NCT04600895	USA	1150	Phase 3
Respiratory system	Cytokine storm	Cytokine inhibitor	Pirfenidone	NCT04856111	India	48	Phase 4
Respiratory system	Cytokine storm	M2 protein inhibitor	Amantadine hydrochloride	NCT04854759	Poland	200	Phase 3
Respiratory system	Cytokine storm	Convalescent plasma	Convalescent plasma	NCT04558476	Belgium	500	Phase 2
Respiratory system	Cytokine storm, vascular endothelial injury	VEGF inhibitor	Nintedanib 150 MG [Ofev]	NCT04541680	France	250	Phase 3
Respiratory system	Host viral entry	Acetylcholine agonists	Nicotine	NCT04608201	France	220	Phase 3
Respiratory system	Cytokine storm	Interferon beta-1a	SNG001	NCT04732949	USA	610	Phase 3
Respiratory system	Cytokine storm	SSRI	Fluvoxamine	NCT04668950	USA	1100	Phase 3
Lung and coagulation system	Host viral entry and higher hypercoagulability	Protease TMPRSS2 inhibition	Nafamostat mesilate, placebo	NCT04352400	Italy	256	Phase 3
Respiratory system	Cytokine storm	IL-6-blocking antibodies	Clazakizumab, placebo	NCT04343989	USA	180	Phase 2
Nervous system	Cytokine storm, neuroinflammation	Endogenous molecule	mPEA and umPEA	NCT04568876	Italy	40	Phase 4
Central nervous system	Cytokine storm, sedatives needed	Sedation drugs	Isoflurane inhalant product, sevoflurane inhalant product	NCT04415060	Canada	752	Phase 3
Central Nervous System	Cytokine storm, sedatives needed	Anti-adrenergic medications	Propranolol hydrochloride	NCT04467086	Canada	108	Phase 3
Neuropsychological system	Cytokine storm, post-viral fatigue syndrome	C1 inhibitor	Ruconest	NCT04705831	USA	40	Phase 4
Peripheral nervous system	Cytokine storm, Nasopharyngitis	Phyto preparation	BNO 1030	NCT04797936	Ukraine	133	Phase 4
Central nervous system	Cytokine storm	Analgesics	Cannabis, medical	NCT03944447	USA	200000	Phase 2
Central nervous system	Cytokine storm, acute brain damage	Surgery	Sphenopalatine ganglion block with local anesthetic	NCT04636034	Denmark	60	Phase 3
Neuropsychiatric system	Viral growth, Cytokine storm	ACE2 inhibitor	Hydroxychloroquine, apixaban	NCT04788355	Brazil	176	Phase 3
Neuropsychiatric system	Cytokine storm, brain damage	Nicotinamide riboside	Niagen	NCT04809974	USA	100	Phase 4
Respiratory system, Neuropsychiatric system	Cytokine storm	IL-6 blocker	Fluoxetine	NCT04377308	USA	2000	Phase 4
Central Nervous System	Cytokine storm	CB1 and CB2 agonists	Cannabidiol	NCT04467918	Brazil	100	Phase 2,3
Neuropsychiatric system	Cytokine storm, brain damage	NMDA inhibitor	Ketamine	NCT04769297	USA	30	Phase 4
Kidney	Cytokine storm	Hormone replacement therapy	Extracorporeal mesenchymal stromal cell therapy (SBI-101 Therapy)	NCT04445220	United States	22	Phase 1, Phase 2
Kidney	Cytokine storm	Hormone replacement therapy	AN-69 Oxiris membrane or the standard AN-69 membrane	NCT04597034	Mexico	35	−
Kidney	AKI,ARDS and COVID-19	Cytopheretic device	Device: SCD	NCT04395911	United States	22	−
Kidney	Thrombotic microangiopathy	C5a inhibitor	Ravulizumab	NCT04570397	United States	32	Phase 3
Kidney	Host viral entry	Urine alkalinisation to prevent binding of SARS-COV-2 to renal tubular epithelial cells	Sodium Bicarbonate 150Meq/L/D5W Inj	NCT04655716	United Kingdom	80	Phase 3
Kidney	Acute kidney injury	Renal protection	Nicotinamide riboside	NCT04818216	United States	100	Phase 2
Kidney	Acute kidney injury	extracorporeal CO2 removal	Device: extracorporeal CO2 removal (ECCO2R) therapy	NCT04351906	Germany	20	−
Kidney	Sepsis, severe acute kidney injury, COVID-19	Plasma expansion with Ringer’s Acetate	7,5 ml/kg mL Ringer’s acetate	NCT02765191	Sweden	20	−
Gastrointestinal	Minimize/avoid any immune response	Immune response	inhalable hydroxychloroquine (HCQ) supportive and symptomatic treatment	NCT04477083	Egypt	40	−
Gastrointestinal	/	/	Hydroxychloroquine	NCT04351620	United States	20	Phase 1
Gastrointestinal	/	/	No intervention	NCT04401124	China	500	−
Gastrointestinal	Gut microbes	Gut microbes	Omni-Biotic Pro Vi 5	NCT04813718	Austria	20	−
Gastrointestinal	/	/	Swallowing evaluation with the EAT-10 and the volume-viscosity swallowing test (V-VST)	NCT04346212	Spain	300	−
Gastrointestinal	/	/	/	NCT04838834	United States	472	−
Gastrointestinal	/	/	Laparoscopic appendectomy	NCT04786041	Israel	200	−
Endocrine system	Uncontrolled blood glucose	Antiviral drug combined with an anti-inflammatory	Drug: Baricitinib Drug: Dexamethasone Drug: Remdesivir	NCT04970719	Bengal	382	Phase 3
Endocrine system	β-cell function	Insulinotropic amino acids	Stimulation test with arginine infusion in order to verify the possible existence of damage to the beta cell function induced by COVID-19 infection	NCT04463849	Italy	90	−
Endocrine system	Cytokine storm	Decrease blood sugar; increase ACE2 expression	Drug: Pioglitazone 30 mg	NCT04535700	Spain	76	Phase 4
Endocrine system	Interleukin-1 (IL-1) beta system	blocking IL-1beta activity	Drug: Canakinumab	NCT04510493	Switzerland	116	Phase 3
Endocrine system	Decrease in TNF-alpha, interleukin, hs CRP, leptin and other inflammatory markers	Anti-inflammatory and inflammation-resolving	Drug: Pioglitazone 45 mg	NCT04604223	Kuwait	1506	Phase 4
Endocrine system	Decrease blood sugar	Glucokinase (GK; hexokinase 4) activator	Drug: AZD1656	NCT04516759	Czech Republic	156	Phase 2
Endocrine system	Decrease blood sugar	Dipeptidyl Peptidase-4 Inhibitor (DPP4i)	Drug: Linagliptin tablet	NCT04542213	Mexico	70	Phase 3
Endocrine system	Cytokine storm	Regulate immune function	Drug: cholecalciferol	NCT04733625	Egypt	56	−
ENT	Cytokine storm	Nasal steroid	Ophtamesone	NCT04569825	Iraq	250	Phase 1
Pre-exposure phrophylaxis	Host viral entry	Pre-exposure phrophylaxis	Truvada	NCT04334928	Spain	1002	Phase 3

ACE2 and TMPRSS2 have been characterized as possible host targets to block SARS-CoV-2 from entering host cells. It is reported that a designed peptide inhibiting SARS-CoV-2 binding to ACE2 and may theoretically block SARS-CoV-2 infection.^410^ Recombinant human ACE2 protein and anti-spike monoclonal antibody could inhibit SARS-CoV-2 S protein-induced platelet activation. MicroRNA molecules targeting ACE2 may be exploited to regulate the SARS-CoV-2 receptor. Administration of microRNA 200c inhibits both ACE2 mRNA and ACE2 protein levels in human iPSC-derived cardiomyocytes and primary cardiomyocytes of COVID-19 rat model, which is a potential regimen for cardiovascular complications of COVID-19.^411^ Besides, excessive ACE2 may competitively bind with SARS-CoV-2, thereby neutralizing the virus and rescuing cellular ACE2 activity which negatively regulates the RAS to protect the lung.^410,412,413^ Therefore, treatment with a soluble form of ACE2 may be effective against SARS-CoV-2 infection. Treatment with anti-androgenic drugs reduced ACE2 expression and protected hESC-derived lung organoids against SARS-CoV-2 infection. Umifenovir, trade name Arbidol has been used to treat COVID-19 in China. The primary mode of action of umifenovir is to inhibit viral attachment by binding to envelope protein.^414^ Camostat mesylate, an orally available serine protease inhibitor, is a potent inhibitor of TMPRSS2 and has been hypothesized as a potential antiviral drug against COVID-19, by inhibiting virus-cell membrane fusion and hence SARS-CoV-2 replication.^259,415,416^ Nafamostat mesylate, which is FDA-approved for indications unrelated to coronavirus infection, inhibits viral entry with roughly 15-fold higher efficiency than camostat mesylate, but requires intravenous dosing.^417^

Chloroquine and hydroxychloroquine may elevate endosomal pH and hinder viral entry and RNA release process.^418^ However, two randomized controlled trials, RECOVERY^419^ and WHO SOLIDARITY trials,^420^ confirmed that these regimens failed to provide any clinical benefit for COVID-19 patients.

Nsps participate in various steps of virus life cycle, including RNA transcription and translation, protein synthesis, processing and modification, virus replication and infection. Among these, nsp5 3CLpro, nsp3 PLpro, nsp12 RdRp and helicase are the most important targets for the development of small-molecule inhibitors because of their biological functions and vital enzyme active site.^421^ The protein sequence similarity between SARS-CoV-2 and SARS-CoV RdRp is up to 96%.^422^ Thus, broad-spectrum antiviral drugs acting on RdRp including nucleoside analogs, e.g., remdesivir, favipiravir, and molnupiravir, may potentially block SARS-CoV-2 replication.^423,424^ ACTT-1 study of intravenous remdesivir in adults who were hospitalized with COVID-19 showed that remdesivir was superior to placebo in shortening recovery time.^425^ However, DisCoVeRy study demonstrated no clinical benefit of remdesivir use in patients hospitalized for COVID-19 who were symptomatic for more than 7 days, and required oxygen support.^426^ Remdesivir undergoes intracellular activation to form an analog of adenosine triphosphate GS-443902 that selectively inhibits viral RNA polymerases and has broad-spectrum activity against coronavirus.^427^ In an animal model molnupiravir is orally active against SARS-CoV-2, and preliminary data of phase 2a trial showed that molnupiravir is highly effective at reducing SARS-CoV-2 RNA and has a favorable safety and tolerability profile.^428^ Protease inhibitors targeting viral 3CLpro are attractive therapeutic options for COVID-19.^429^ Protease inhibitor lopinavir–ritonavir showed significant inhibitory effects on SARS-CoV-2 in vitro.^430^ However, the LOTUS^294^ and RECOVERY^431^ clinical trials independently showed no benefit of using lopinavir–ritonavir in reducing mortality rate, hospital time nor progression to mechanical ventilator intervention. There are three SARS-CoV-2 virulence factors nsp1, nsp3c, and ORF7a related to interfering host’s innate immunity and assisting immune escape, suggesting that nsp1, nsp3c, and ORF7a may be potential targets for antiviral drug development.^421,432^ The efficacy of existing antiviral ribonucleoside and ribonucleotide analogs, such as remdesivir, can be decreased by the viral proofreading exonuclease nsp14-nsp10 complex. Nsp14-nsp10 inhibitors were identified that increase antiviral potency of remdesivir. A model compound, sofalcone, inhibits the exonuclease activity of SARS-CoV-2 in vitro, and synergistically enhances the antiviral effect of remdesivir.^433^ Nsp3 in SARS-CoV-2 serves to counteract the antiviral function of host Poly ADP-ribose polymerase (PARP) which is NAD^+^-consuming enzymes. Therefore, NAD^+^ and NAD^+^-consuming enzymes play crucial roles in immune responses against viral infection. Thorough mechanistic understandings of SARS-CoV-2 replication will likely facilitate the development of general antiviral strategies.^434^

A minimally pathogenic human betacoronavirus (OC43) was used to infect physiologically-relevant human pulmonary fibroblasts MRC5 to facilitate rapid antiviral discovery in a preclinical model. Several FDA-approved agents that can attenuate both OC43 and SARS-CoV-2 viral replication, including lapatinib, doramapimod, and tanespimycin. Importantly, lapatinib inhibited SARS-CoV-2 RNA replication by over 50,000-fold. Further, both lapatinib and doramapimod could be combined with remdesivir to improve antiviral activity in cells. These findings reveal novel therapeutic avenues that could limit SARS-CoV-2 infection.^435^

The knowledge accumulated to date indicates that COVID-19 severity and the associated mortality rate derive either from a dysregulated immunopathology induced directly by SARS-CoV-2 infection or by the tissue damage caused by the immune response against SARS-CoV-2.^15^ Therefore, the altered immune response represents the important target for therapeutic interventions aimed at modifying the immunopathogenesis of COVID-19. Targeting the specific COVID-19 immune profiles, such as by inhibiting inflammation or enhancing lymphocytes are promising treatment strategies for severe cases. Targeting cytokine storm and the signaling pathways have been considered as potentially effective strategies to modulate the hyperinflammatory response against SARS-CoV-2 infection.^436^ Anti-cytokine therapy such as IL-6, TNF-α and IL-1 antagonists have been suggested for the alleviation of hyperinflammation.^357^ In hospitalized COVID-19 patients with hypoxia and systemic inflammation, IL-6R antagonist tocilizumab improved survival and other clinical outcomes.^437,438^ However, a randomized trial in patients with severe or critical COVID-19, tocilizumab failed to improve clinical outcomes, and it might increase mortality.^439^ A randomized, double-blind trial did not show efficacy of another IL-6R antagonist sarilumab in patients admitted to hospital with COVID-19 and receiving supplemental oxygen.^440^ Anti-IL-6 monoclonal antibodies such as siltuximab and sirukumab are under investigation for COVID-19 patients. Aberrant high GM-CSF levels have been detected in circulating lymphocyte populations, excluding NKs and B cells, from patients with COVID-19 admitted to ICU.^78^ Therefore, the potential of GM-CSF-blocking antibodies such as lenzilumab (LIVE-AIR study) and gimsilumab to treat COVID-19 is being evaluated by researchers and pharmaceutical companies.^441^

Other possible strategies under clinical and preclinical investigation for inhibiting macrophage activation include the blockade of certain cytokines, inhibition of C-C chemokine receptor type 5 (CCR5)-mediated migration and CD14 blockade by monoclonal antibodies.^441^ Strategies such as MSC-based therapy, Treg-based therapy and blood purification may also represent alternative effective approach to alleviating SARS-CoV-2-related immunopathology.^15^ SARS-CoV-2 has demonstrated to induce apoptosis of circulating lymphocytes by P53 activation.^442^ T cells from COVID-19 patients expressed higher levels of the exhausted marker PD-1. Increasing PD-1 expression on T cells was observed as disease progressed.^97^ The efficacy of PD-1 monoclonal antibody, camrelizumab plus thymosin have been evaluated in a clinical trial for COVID-19 treatment.^443^ Circulating NK cell numbers were found significantly reduced in COVID-19 patients with severe disease,^444^ and showed increased expression of inhibitory receptor TIM-3.^445^ Chimeric antigen receptor (CAR) -engineered NK cells are also being tested for treating COVID-19.^446^

PAI-1 inhibitors significantly enhance the bronchoalveolar fibrinolytic system and relieve symptoms of COVID-19 by increasing fibrinolytic protein levels that effectively remove fibrin.^447^ There is increasing evidence that complement is involved in SARS-CoV-2 pathology, and that complement inhibitors may reduce the severity of COVID-19 complications and the number of intensive care or deaths, especially with ekuzumab showing preliminary efficacy.^448^ Defibrotide can counteract endothelial activation and hypercoagulability induced by NETs and histone H4, promote endothelial remodeling and prevent endothelial dysfunction.^449^ Blocking vascular endothelial growth factor (VEGF) and VEGF receptor -mediated signaling improves oxygen perfusion and anti-inflammatory responses, and reduces clinical symptoms in patients with severe COVID-19. A humanized monoclonal antibody against VEGF, Bevacizumab plus standard care can be very beneficial for patients with severe COVID-19.^450^ The lung function of COVID-19 patients improved significantly after FX06 administration, which may be attributed to its immunomodulatory properties and its ability to protect the endothelial barrier and reduce vascular hypertonicity.^451^

Given hypercoagulability was commonly seen in patients with COVID-19, studies suggest that low-molecular weight heparin (LMWH) should be used for early and long-term drug-induced thrombosis prevention.^452^ Consistently, in a cohort of critically ill COVID-19 patients with a high prevalence of thromboembolic events, enhanced thromboprophylaxis was associated with reduced ICU mortality without an increased hemorrhagic risk.^453^ However, results from some other multicenter studies did not support routine empirical use of prophylactic anticoagulation in patients with COVID-19.^454–456^ A retrospective analysis indicated that therapeutic anticoagulation was associated with lower mortality among hospitalized COVID-19 patients compared with prophylactic anticoagulation, although not statistically significant.^457^ While two other studies showed that therapeutic anticoagulation did not significantly improve the prognosis nor increase the risk of bleeding compared with prophylactic anticoagulation in patients hospitalized with COVID-19 and elevated D-dimer concentration.^458,459^ A study in France involving 10 patients with ischemic stroke caused by macrovascular embolism showed that early intravenous thrombolysis and mechanical thrombectomy recanalization did not reverse the adverse outcomes of patients.^460^ These controversial results demonstrate the complexity of coagulation in COVID-19 patients.

To date, the research on the mechanism of COVID-19 related coagulation disorders has proposed a series of molecules and pathways that may be used as clinical intervention targets. First, disorders in the coagulation system include colocalization of coagulation factor XII and NETs,^153^ increased biological function of CD142 which exposed onto surface of cell-released extracellular vesicles,^461,462^ overactivation of the complement component anaphylatoxin-NET axis,^462^ overactivation of platelet via binding to S protein by ACE2.^145^ Studies shows that by targeting these molecules will improve the coagulation state in vitro. Second, a reduction in fibrinolysis also plays an important role in COVID-19-associated coagulopathy, and promoting fibrinolytic activity is a possible way to change the coagulation disorder in patients.^463–465^ Third, studies have found that vascular endothelial cells activation and dysfunction mediate inflammation and abnormal coagulation in COVID-19 patients.^106,107^ In addition, some other factors can also play a role by influencing the above three systems, such as RAS,^466^ complement and coagulation cascade signaling,^125,467,468^ mineralocorticoid receptor (MR) and its downstream target galectin-3 (Gal-3),^469^ IL-6,^124^ extrahepatic vitamin K insufficiency,^470^ etc. These molecules and pathways form a complex network leading to the complexity of the disease and the difficulty of the treatment. Some drugs targeting the above molecules and pathways have entered clinical trials, such as inhaled rhDNase1 (targeting the NETs), recombinant bacterial ACE2 receptors-like enzyme of B38-CAP, RAS inhibitor, mineralocorticoid receptor antagonist (MRA) canrenoate potassium, the IL-6 inhibitor clazakizumab, vitamin K2, etc.

Accumulating literature has demonstrated the beneficial effects of n-3 polyunsaturated fatty acids (n-3 PUFA) toward the cardiovascular system, which include ameliorating uncontrolled inflammatory reactions, reduced oxidative stress and mitigating coagulopathy.^471^ Due to the favorable safety profile of n-3 PUFAs and their metabolites, it is reasonable to consider n-3 PUFAs as potential adjuvant therapies for the clinical management of COVID-19 patients. Targeting RAGE to prevent SARS-CoV-2-mediated multiple organ failure is also a promising therapy.^472^ Pharmacological agents frequently used in atherosclerotic conditions, such as statins and aspirin, appear to lower the incidence of serious COVID-19 complications and mortality rates.^473^ Oxytocin (OXT) can protect the heart and vasculature through suppressing hypertension and brain-heart syndrome, and promoting regeneration of injured cardiomyocytes.^474^ Exogenous OXT can be used safely without the side-effects seen in remdesivir and corticosteroid.^474^

A retrospective observational study of COVID-19 patients with type 2 diabetes found that sitagliptin treatment during hospitalization was associated with reduced mortality and improved clinical outcomes.^475^ Evidence suggests that insulin and dipeptidyl peptidase-4 (DDP4) inhibitors can be used safely in COVID-19 patients with diabetes. Metformin and sodium-glucose cotransporter-2 (SGLT-2) inhibitors might need to be withdrawn in patients at high risk of severe disease.^476^

## SARS-CoV-2 vaccination

The vaccination of SARS-CoV-2 vaccine may become one of the most effective means to terminate COVID-19 epidemic. The current vaccine development mainly uses viral S protein, S protein receptor-binding domain, or S protein subunits as antigens. The technical strategies^477^ include: viral vaccines (live attenuated vaccines and inactivated vaccines), viral-vectored vaccines (replicating and non-replicating), nucleic acid vaccines (DNA vaccines and mRNA vaccines), protein subunit vaccines (recombinant protein vaccines, protein subunit vaccines and virus-like particle vaccines). As of January 7, 2022, 137 of the 331 vaccine projects announced by the WHO^478^ have entered the clinical trial stage. At present, several vaccines produced in China, the United States and Europe have been the first to vaccinate people on a large scale. However, the main challenge is the reduction of the protective power of the vaccine due to the mutation of the SARS-CoV-2. Efforts to develop polyvalent vaccines^479^ against different strains may be a solution, but the mutation of SARS-CoV-2 is rapid which makes the development of vaccine extremely difficult. Another option is to develop oral and spray vaccines.^480^ The advantage lies in that it can increase the mucosal immune response and improve the effectiveness of neutralizing antibodies; low-temperature storage is no longer required, and the transportation problem is solved, thereby facilitating use and promotion. A phase I clinical study^481^ showed that nebulization of one dose of the spray type vaccine Ad5-nCoV required only 1/5 of the dose for intramuscular injection, and nebulization of two doses of Ad5-nCoV produced antibody and cellular immune responses comparable to that of a single dose of intramuscular injection of this vaccine. Moreover, high levels of neutralizing antibodies can be produced by booster immunization using nebulization after intramuscular injection.

With the emergence of the delta variant, the third dose of vaccine booster has been implemented in various countries. In Israel, people who have been vaccinated with two doses of BNT162b2 vaccine for about 8 months received a third dose of BNT162b2 vaccine. It was found that the neutralization geometric mean titer (GMT) for the β variant increased more than the GMT for the wild-type virus,^482^ and the adverse events did not increase significantly. At least 12 days after the booster vaccination, the confirmed infection rate of the boosted group was 11.3 times lower than that of the unboosted group; the severe disease rate was reduced by 19.5 times.^483^ Preliminary data showed that the same three doses of inactivated virus vaccine can also enhance and maintain the immune response, and the peak antibody level is about 25 times higher than before the injection. Even six months after the injection, the antibody level is still comparable to the peak after the second dose. The above-mentioned researches focus on the effect of homologous prime-boost vaccinations, while heterologous prime-boost immunization strategies have also been under investigation and preliminary results demonstrate a significant increase in the level of neutralizing antibody.

## Summary

In addition to pneumonia and ARDS, severe COVID-19 mainly involves multiple extrapulmonary organs and systems such as cardiovascular, renal, gastrointestinal, and hepatobiliary systems, as well as hematological, neurological, endocrine and metabolic systems, etc. SARS-CoV-2 may directly invade the host cells of multiple organs through the ACE2 that is widely distributed in various human tissues and TMPRSS2 or other possible entry routes. Moreover, cytokine storm and infiltration of inflammatory cells, dysregulated immune responses, coagulation dysfunction, and epithelial injury can induce multiorgan failure in the severe cases with COVID-19. Gaining a whole picture of the clinical features of multiorgan dysfunction in critically ill patients with COVID-19 is of highly great importance for both clinicians and researchers. Consequently, fulfilling the knowledge on the potential mechanisms underlying of SARS-CoV-2-induced pulmonary and extrapulmonary complications may ultimately lead to the development of potential therapeutic approaches for COVID-19, which will eventually eradicate COVID-19 across the globe. Nevertheless, given the rapidly evolving scenario due to the emergence of SARS-CoV-2 variants and ongoing vaccination campaigns, more studies are warranted to achieve comprehensive knowledge of the multifaceted interaction between the host and SARS-CoV-2.
